# Orexin 2 receptor–selective agonist danavorexton improves narcolepsy phenotype in a mouse model and in human patients

**DOI:** 10.1073/pnas.2207531119

**Published:** 2022-08-22

**Authors:** Rebecca Evans, Haruhide Kimura, Robert Alexander, Ceri H. Davies, Hélène Faessel, Deborah S. Hartman, Takashi Ishikawa, Emiliangelo Ratti, Kohei Shimizu, Motohisa Suzuki, Shinichiro Tanaka, Hiroshi Yukitake, Yves Dauvilliers, Emmanuel Mignot

**Affiliations:** ^a^Neuroscience Therapeutic Area Unit, Takeda Pharmaceuticals International Co., Cambridge, MA 02139;; ^b^Neuroscience Drug Discovery Unit, Research, Takeda Pharmaceutical Company Limited, Fujisawa, 251–8555, Japan;; ^c^Takeda Development Center Japan, Takeda Pharmaceutical Company Limited, Osaka, 532–8686, Japan;; ^d^National Reference Network for Narcolepsy, Sleep-Wake Disorders Center, Department of Neurology, Gui-de-Chauliac Hospital, CHU Montpellier, INM, INSERM, University of Montpellier, Montpellier, 34295, France;; ^e^Stanford Department of Psychiatry and Behavioral Medicine, Center for Sleep Sciences and Medicine, Stanford University Medical School, Palo Alto, CA 94304

**Keywords:** narcolepsy, orexin 2 receptor, agonist, danavorexton

## Abstract

Narcolepsy type 1 (NT1) is a sleep disorder caused by a loss of orexinergic neurons. Narcolepsy type 2 (NT2) is heterogeneous; affected individuals typically have normal orexin levels. Following evaluation in mice, the effects of the orexin 2 receptor (OX2R)-selective agonist danavorexton were evaluated in single- and multiple-rising-dose studies in healthy adults, and in individuals with NT1 and NT2. In orexin/ataxin-3 narcolepsy mice, danavorexton reduced sleep/wakefulness fragmentation and cataplexy-like episodes during the active phase. In humans, danavorexton administered intravenously was well tolerated and was associated with marked improvements in sleep latency in both NT1 and NT2. In individuals with NT1, danavorexton dose-dependently increased sleep latency in the Maintenance of Wakefulness Test, up to the ceiling effect of 40 min, in both the single- and multiple-rising-dose studies. These findings indicate that OX2Rs remain functional despite long-term orexin loss in NT1. OX2R-selective agonists are a promising treatment for both NT1 and NT2.

## Significance

Despite the discovery of the orexin (hypocretin) neuropeptides in 1998 and their role in narcolepsy type 1 (NT1), current pharmacotherapy addresses only the associated symptoms and not the underlying loss of orexin. Danavorexton (TAK-925) is an orexin 2 receptor (OX2R)–selective agonist that was developed to address the loss of orexin signaling in NT1. Here, we present promising results from preclinical mouse studies through to human clinical studies, which support the therapeutic potential of OX2R-selective agonists for the treatment of NT1. Improvements in excessive daytime sleepiness were also observed in individuals with narcolepsy type 2, indicating that OX2R-selective agonists could also provide therapy for sleep disorders that involve partial or no impairment of the orexin system.

Narcolepsy is a chronic disorder of hypersomnolence that is subclassified as narcolepsy type 1 (NT1, narcolepsy with cataplexy; sudden episodes of muscle weakness triggered by emotions) or narcolepsy type 2 (NT2, narcolepsy without cataplexy) (1–3). NT1 is caused by a loss of orexinergic neurons, resulting in low levels of orexin (also called hypocretin) neuropeptides in the brain (4), as measured in the cerebrospinal fluid (CSF; orexin A <110 pg/mL) (5). It is not known whether orexin receptors in individuals with NT1 remain functional, although a postmortem study using tissue from five individuals with NT1 showed no significant change in *HCRTR2* (encoding orexin 2 receptor [OX2R]) messenger RNA expression levels versus controls (6). Although the cause of NT2 remains poorly understood, some cases have been shown to involve partial loss of orexinergic neurons (7, 8) and some to involve intermediate CSF orexin A levels (9, 10). However, in most cases CSF orexin levels are in the normal range (2, 3, 7, 8), suggesting no ligand deficiency, although downstream alterations in orexin signaling cannot be excluded as a possible cause.

Orexinergic neurons in the lateral hypothalamus produce two orexin neuropeptides (orexin A and orexin B), derived from the same precursor polypeptide but with structurally different N-termini, that act on orexin receptors (orexin 1 receptor [OX1R] and OX2R) throughout the central nervous system (11, 12). Sleep and wakefulness are strongly regulated by the orexin system (13), with OX2R activation playing a prominent role in promoting wakefulness (14). Orexin A and B both bind to OX2R with high affinity (12).

Polypharmacy is often needed to address the various symptoms of NT1 (15, 16), but no current treatments directly address the loss of orexin. Given that orexin neuropeptides cannot pass the blood–brain barrier (17), nonpeptide, brain-penetrant orexin agonists are logical potential treatments (14–16, 18). The OX2R-selective agonist danavorexton (TAK-925, [methyl (2*R*,3*S*)-3-[(methylsulfonyl)amino]-2-{[(*cis*-4-phenylcyclohexyl)oxy]methyl}piperidine-1-carboxylate]) was developed to address the loss of orexin signaling in NT1 and has shown >5,000-fold selectivity for human OX2R versus OX1R in vitro. Furthermore, danavorexton induced wakefulness in wild-type (WT) mice but not in OX2R knockout mice (19).

Here, we evaluate the effects of danavorexton further, using a transgenic mouse model of NT1 in which orexinergic neurons are ablated (orexin/ataxin-3 mice) (20). Based on these data, two phase 1 clinical studies (TAK-925–1001 and TAK-925–1003) were designed to translate the effects observed in narcoleptic mice to humans with NT1. TAK-925–1001 was a single-rising-dose (SRD) study in healthy volunteers and adults with NT1 (NCT03332784). TAK-925–1003 was a multiple-rising-dose (MRD) study in healthy volunteers and adults with NT1 or NT2 (NCT03748979). Given that wakefulness-promoting effects were observed in WT mice, patients with NT2 were included to evaluate the effects of OX2R-selective agonists in disorders of hypersomnolence that may or may not involve loss of orexinergic neurons.

## Results

### Effects of Danavorexton in Orexin/Ataxin-3 Mice as a Model of NT1.

#### Effects on sleep/wakefulness fragmentation and cataplexy-like episodes.

**Fig. 1.**
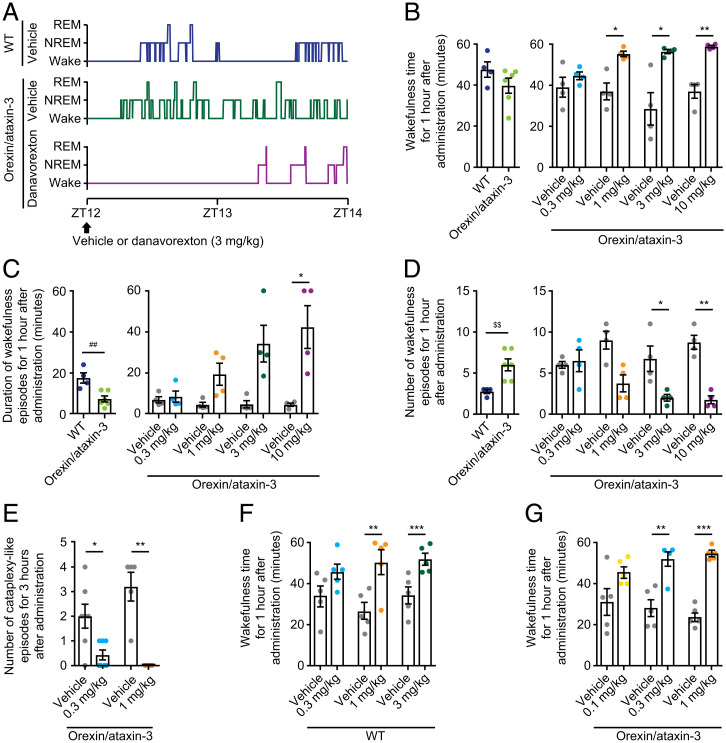
Danavorexton effects on sleep/wakefulness fragmentation and cataplexy-like episodes in orexin/ataxin-3 narcoleptic mice. (*A*) Representative hypnogram for 2 h after danavorexton (3 mg/kg SC) single administration. In orexin/ataxin-3 mice during active phase, effect of danavorexton single doses (0.3, 1, 3, and 10 mg/kg SC) for 1 h after administration on: (*B*) total wakefulness time, (*C*) duration of wakefulness episodes, and (*D*) number of wakefulness episodes. (*E*) In orexin/ataxin-3 mice during active phase, effect of danavorexton single doses (0.3 mg/kg and 1 mg/kg SC) on cataplexy-like episodes. ##*P* ≤ 0.01 (two-tailed Student *t-*test; WT, *n* = 4; orexin/ataxin-3, *n* = 6), $$*P* ≤ 0.01 (two-tailed Aspin–Welch test; WT, *n* = 4; orexin/ataxin-3, *n* = 6), *P ≤ 0.05, **P ≤ 0.01 (two-tailed paired *t* test with closed testing procedure from the high-dose side; [B–D], *n =* 4; [E, 0.3 mg/kg], *n* = 7; [E, 1 mg/kg], *n* = 5). Mean ± SEM. (*F*) Effects of danavorexton on wakefulness time in WT mice (*n* = 5). (*G*) Effects of danavorexton on wakefulness time in orexin/ataxin-3 mice (*n* = 5). Mean ± SEM. **P ≤ 0.01, ***P ≤ 0.001 (two-tailed paired *t* test with closed testing procedure from the high dose side).

Danavorexton administrations increased wakefulness and reduced fragmentation of wakefulness in orexin/ataxin-3 mice during the active/awake phase (Fig. 1*A*). During the first hour after administration, danavorexton at 1, 3, and 10 mg/kg significantly increased total wakefulness time (*P* ≤ 0.05 at 1 and 3 mg/kg, *P* ≤ 0.01 at 10 mg/kg; Fig. 1*B*), an effect associated with a concomitant decrease in total time of non–rapid eye movement (NREM) (*SI Appendix*, Fig. S1*A*) and rapid eye movement (REM) sleep (*SI Appendix*, Fig. S1*D*). Compared with vehicle control, longer durations of wakefulness episodes were observed with danavorexton 10 mg/kg (*P* ≤ 0.05; Fig. 1*C*). Furthermore, fewer wakefulness episodes were observed with danavorexton 3 and 10 mg/kg (*P* ≤ 0.05 and *P* ≤ 0.01, respectively; Fig. 1*D*), showing that danavorexton reduced the fragmentation of wakefulness. As a result, decreases in episode duration and in the number of both NREM (*SI Appendix*, Fig. S1 *B* and *C*) and REM sleep episodes (*SI Appendix*, Fig. S1 *E* and *F*) were observed.

The effects of danavorexton on cataplexy-like episodes were assessed by using chocolate as an exemplar emotional stimulus to trigger cataplexy. In this paradigm, danavorexton 0.3 and 1 mg/kg suppressed cataplexy-like episodes in orexin/ataxin-3 mice during the active phase in the 3 h following administration (*P* ≤ 0.05 and *P* ≤ 0.01, respectively; Fig. 1*E*). These findings were confirmed in a separate group of orexin/ataxin-3 mice with video recording used to confirm immobility during cataplexy-like episodes, in addition to locomotor activity recording, with danavorexton 1 mg/kg (*P* ≤ 0.01; *SI Appendix*, Fig. S1*G*).

Pharmacodynamic (PD) and pharmacokinetic (PK) analysis revealed that danavorexton induced a potent, sustained wakefulness-promoting effect when plasma concentrations exceeded a threshold concentration of ∼100 ng/mL in orexin/ataxin-3 mice (*SI Appendix*, Fig. S2 *A* and *B*). Observation of this threshold effect guided the study design and dosage selection for NT1 cohorts in the human study using intravenous (IV) infusion.

#### Orexin/ataxin-3 narcoleptic mice are hypersensitive to danavorexton.

Analyses were undertaken to assess potential changes in sensitivity to an OX2R-selective agonist following chronic loss of orexin, as observed in patients with NT1. In WT mice during their sleep/inactive phase, danavorexton 1 and 3 mg/kg significantly increased total wakefulness time in the first hour after administration (Fig. 1*F*), an effect associated with a decrease in total NREM sleep time (danavorexton 1 mg/kg, *P* ≤ 0.01; danavorexton 3 mg/kg, *P* ≤ 0.001) (*SI Appendix*, Fig. S3*A*) and a trend toward decreased total REM sleep time (*SI Appendix*, Fig. S3*B*) when compared with vehicle. In orexin/ataxin-3 mice during their sleep/inactive phase, danavorexton 0.3 and 1 mg/kg significantly increased total wakefulness time during the first hour after administration (Fig. 1*G*), decreased total NREM sleep time (danavorexton 0.3 mg/kg, *P* ≤ 0.01; danavorexton 1 mg/kg, *P* ≤ 0.001) (*SI Appendix*, Fig. S3*C*), and did not affect total REM sleep time (*SI Appendix*, Fig. S3*D*) when compared with vehicle.

Orexin/ataxin-3 mice thus appeared to have a threefold higher sensitivity for danavorexton than WT mice, with minimum effective dosages of danavorexton 0.3 mg/kg and 1 mg/kg in orexin/ataxin-3 mice and WT mice, respectively.

#### Effects of danavorexton chronic dosing.

**Fig. 2.**
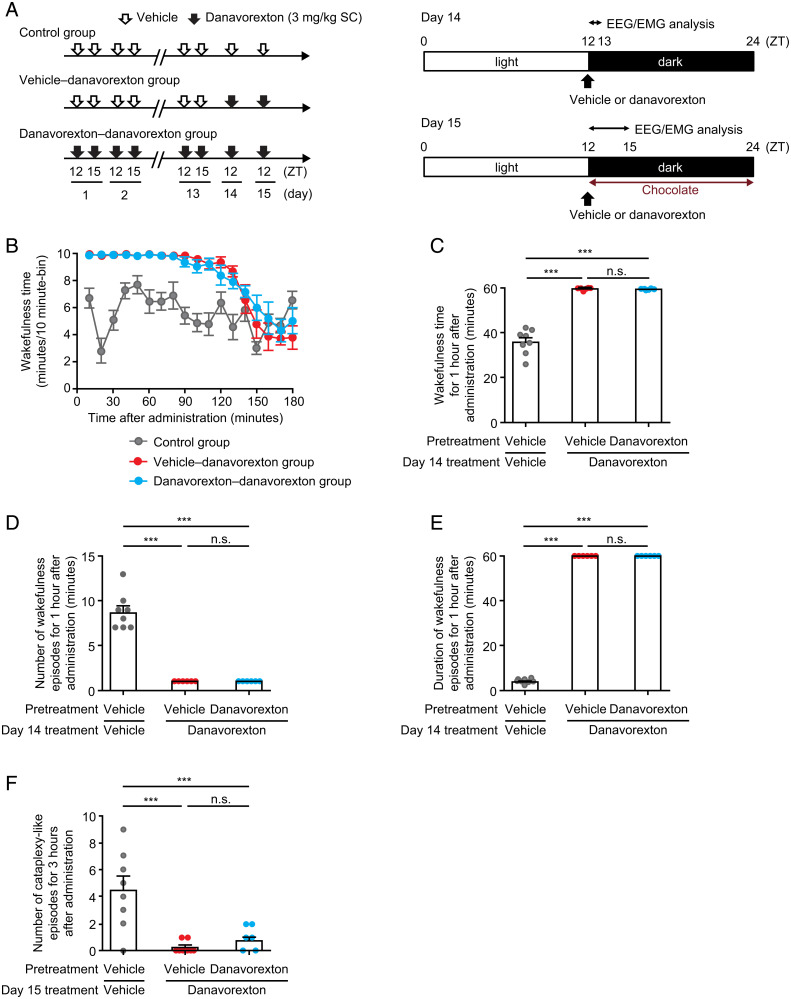
Effects of danavorexton chronic dosing in orexin/ataxin-3 narcoleptic mice. Danavorexton increased wakefulness and ameliorated cataplexy-like episodes in orexin/ataxin-3 mice during the active phase after both single and repeated administration. (*A*) Time schedule of drug administration in mice during the active phase. (*B*) Time course of wakefulness after vehicle or danavorexton administration in orexin/ataxin-3 mice on day 14. In orexin/ataxin-3 mice during the active phase on day 14 (*n* = 8), effects of single and repeated administration of danavorexton on: (*C*) wakefulness time, (*D*) number of wakefulness episodes, and (*E*) duration of wakefulness episode. (*F*) In orexin/ataxin-3 mice during the active phase on day 15 (*n* = 8), effects of single and repeated administration of danavorexton on cataplexy-like episodes. Mean ± SEM. ***P ≤ 0.001 (two-tailed Tukey’s test). n.s., not significant; ZT, zeitgeber time.

On day 14, following pretreatment with danavorexton 3 mg/kg or vehicle for 13 d, danavorexton 3 mg/kg or vehicle was administered at zeitgeber time 12 (i.e., at the start of the 12-h dark cycle), followed by electroencephalographic and electromyographic recording (Fig. 2*A*). The time course of wakefulness on day 14 was similar between mice pretreated with danavorexton 3 mg/kg and those pretreated with vehicle only, indicating that chronic pretreatment with danavorexton did not change the threshold concentration for wakefulness efficacy in these experimental conditions (Fig. 2*B*).

In line with preserved threshold effective concentrations, both single and repeated administration of danavorexton 3 mg/kg significantly increased wakefulness time when compared with vehicle in orexin/ataxin-3 mice during the first hour after administration in their active phase on day 14 (both *P* ≤ 0.001) (Fig. 2*C*). Concomitantly, total NREM and REM sleep times (*SI Appendix*, Fig. S4) were decreased after both single and repeated administration of danavorexton 3 mg/kg versus vehicle during the first hour after administration (both *P*s ≤ 0.001). Reduced fragmentation of wakefulness was observed with both single and repeated administration, with danavorexton 3 mg/kg significantly reducing the number of wakefulness episodes and increasing the duration of each episode (both *P*s ≤ 0.001) when compared with vehicle (Fig. 2 *D* and *E*).

On day 15, the effect of danavorexton on cataplexy-like episodes in orexin/ataxin-3 mice was reevaluated following presentation of chocolate (Fig. 2*A*). Both single and repeated administration of danavorexton 3 mg/kg reduced the number of cataplexy-like episodes for 3 h after administration (*P* ≤ 0.001) when compared with vehicle (Fig. 2*F*).

There was no statistical difference in wakefulness time, sleep/wakefulness fragmentation, or cataplexy-like episodes between single and repeated administrations of danavorexton.

### SRD and MRD Clinical Studies TAK-925–1001 and TAK-925–1003.

#### Study populations.

In both studies, safety and efficacy analysis sets were defined as all participants who received at least one dose of study drug. In the SRD study, the safety analysis set comprised 58 participants: 36 healthy adults (median [range] age, 24.5 [20–40] years; 36 men), including a subset of four participants included in the CSF analysis (median [range] age, 20 [21–22] years); eight healthy older adults (median [range] age, 68 [65–75] years; four men); and 14 individuals with NT1 (median [range] age, 32.5 [18–59] years; six men). Healthy volunteers received danavorexton doses of 7–240 mg, and individuals with NT1 received doses of 5, 11.2, or 44.8 mg (*SI Appendix*, Tables S1 and S2). At baseline, adults with NT1 had a slightly higher mean body mass index (BMI) than healthy volunteers.

In the MRD study, the safety analysis set comprised 24 healthy adults (median [range] age, 22 [20–30] years; 24 men), 13 individuals with NT1 (median [range] age, 32 [18–42] years; eight men), and 14 individuals with NT2 (median [range] age, 24.5 [20–48] years; six men) (*SI Appendix*, Table S3). Healthy volunteers received danavorexton doses of 44, 112, or 180 mg, whereas individuals with NT1 received doses of 11 mg or 44 mg, and individuals with NT2 received doses of 44 mg or 112 mg (*SI Appendix*, Table S3).

The baseline NT1 symptom severity profile was similar in study participants of the SRD and MRD studies (*SI Appendix*, Tables S2 and S3).

#### Maintenance of Wakefulness Test.

**Fig. 3.**
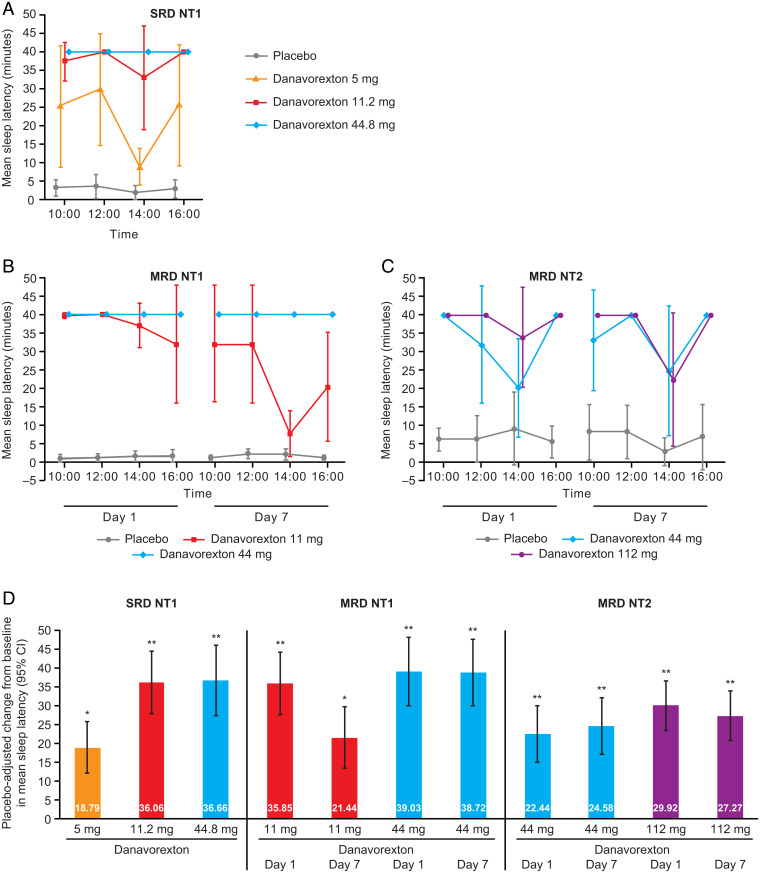
Nine-hour intravenous infusions of danavorexton improved Maintenance of Wakefulness Test sleep latency in participants with NT1 and NT2. Mean sleep latency in each MWT session in: (*A*) participants with NT1 in the SRD study, (*B*) participants with NT1 in the MRD study, and (*C*) participants with NT2 in the MRD study. (*D*) Placebo-adjusted change from baseline in the overall mean of all four MWT sessions in participants with NT1 in the SRD study and in participants with NT1 or NT2 in the MRD study (day 1 and day 7). Error bars in (*A*–*C*) indicate SD. Error bars in (*D*) indicate 95% CI. SRD study: analysis of covariance of mean sleep latency, *P < 0.001, **P < 0.0001. MRD study: mixed model for repeated measures analysis of mean sleep latency, *P < 0.001, **P < 0.0001.

In the SRD study, danavorexton dose-dependently increased wakefulness in individuals with NT1, as measured by time to sleep onset in the Maintenance of Wakefulness Test (MWT) (Fig. 3*A*). Overall, the placebo-adjusted change in average sleep latency (95% CI) was 18.79 (11.99, 25.60) minutes for danavorexton 5 mg, 36.06 (27.73, 44.40) minutes for danavorexton 11.2 mg and 36.66 (27.34, 45.98) minutes for danavorexton 44.8 mg (Fig. 3*D*).

Similar results were observed in the MRD study of individuals with NT1, with all those who received the higher dose of 44 mg scoring the maximum sleep latency of 40 min in all MWT sessions on days 1 and 7 (Fig. 3*B*). Compared with placebo, on day 1, the overall average sleep latency improved from baseline by 35.85 min with danavorexton 11 mg (95% CI, 27.66–44.04; *P* < 0.0001) and 39.03 min with danavorexton 44 mg (95% CI, 29.85–48.20; *P* < 0.0001). On day 7, the overall average placebo-adjusted sleep latency improved from baseline by 21.44 min with danavorexton 11 mg (95% CI, 13.26–29.63; *P* = 0.0002) and 38.72 min with danavorexton 44 mg (95% CI, 29.95–47.50; *P* < 0.0001) (Fig. 3*D*).

In individuals with NT2, both doses of danavorexton increased sleep latency compared with placebo (Fig. 3*C*). Compared with placebo, on day 1 the overall average sleep latency improved from baseline by 22.44 min with danavorexton 44 mg (95% CI, 14.88–30.00; *P* < 0.0001) and 29.92 min with danavorexton 112 mg (95% CI, 23.23–36.61; *P* < 0.0001). On day 7, the overall average placebo-adjusted sleep latency improved from baseline by 24.58 min with danavorexton 44 mg (95% CI, 17.02–32.14; *P* < 0.0001) and 27.27 min with danavorexton 112 mg (95% CI, 20.58–33.96; *P* < 0.0001) (Fig. 3*D*).

#### Epworth Sleepiness Scale.

**Fig. 4.**
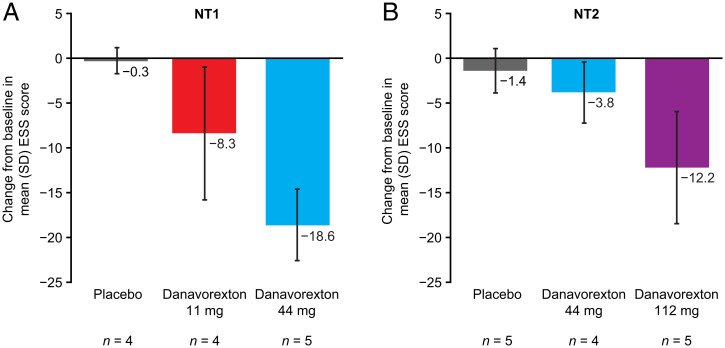
Mean change in Epworth Sleepiness Scale score from baseline to day 7 in the multiple-rising-dose study. Error bars indicate SD.

Participants in the MRD study were assessed via the Epworth Sleepiness Scale (ESS) on day –1 and day 7, and were asked to consider the past 7 d when completing the survey. Danavorexton substantially improved subjective sleepiness in participants with NT1, as indicated by the mean change from baseline on day 7 (Fig. 4). Baseline mean ESS values for participants with NT1 were 16.5 (SD, 4.65), 18.0 (SD, 4.69), and 18.6 (SD, 4.04) in the placebo, danavorexton 11 mg, and danavorexton 44 mg groups, respectively. On day 7, these scores fell to within the normal range of 0–10 for both danavorexton 11 mg (9.8 [SD, 3.59]) and danavorexton 44 mg (0.0 [SD, 0.00]). Baseline mean ESS values for participants with NT2 were 17.6 (SD, 3.05), 17.0 (SD, 2.16), and 18.2 (SD, 2.17) in the placebo, danavorexton 44 mg, and 112 mg groups, respectively. On day 7, these scores fell to 13.3 (SD, 5.62) with danavorexton 44 mg and 6.0 (SD, 5.83) with danavorexton 112 mg.

#### Karolinska Sleepiness Scale.

Over the 9-h study drug infusion period in the SRD study, individuals with NT1 had lower Karolinska Sleepiness Scale (KSS) ratings (indicating greater alertness) in all danavorexton dose groups than in the pooled placebo group at all time points assessed. Mean change from baseline to 9 h after the start of infusion in KSS score was 0.3 (SD, 2.87) for placebo, −2.5 (SD, 1.52) for danavorexton 5 mg, and −1.3 for both danavorexton 11.2 mg (SD, 2.87) and danavorexton 44.8 mg (SD, 1.50).

In the MRD study, on day 1 and day 7, improvements on the KSS were seen with danavorexton 44 mg but not with 11 mg in individuals with NT1. In individuals with NT2, no significant differences from placebo were observed with either danavorexton 44 mg or 112 mg (Table 1).

**Table 1.** Change from baseline to 9 h after the start of infusion in KSS, PVT, and PGI-C in individuals with NT1 and individuals with NT2 in the MRD study.

Adults with NT1					
	Placebo (*n* = 4)	Danavorexton 11 mg (*n* = 4)	*P* value 11 mg versus placebo	Danavorexton 44 mg (*n* = 5)	*P* value 44 mg versus placebo
**KSS** *					
Day 1	–0.9	–2.7	0.0174	–3.9	0.0006
Day 7	–1.2	–1.7	0.4272	–4.1	0.0007
**PVT** ^†^ ** (number of lapses)**					
Day 1	11.5	–12.1	0.0086	–13.4	0.0059
Day 7	12.7	–14.9	0.0032	–13.4	0.0045
**PVT** ^†^ ** (change in mean duration of the reciprocal 10% slowest reaction time [1/RT])**					
Day 1	−0.36	0.72	0.0052	0.59	0.0062
Day 7	−0.59	0.98	0.0004	0.78	0.0007
**PGI-C scale (reported 9 h after the start of infusion on day 7), *n* (%)**					
Very much improved	0	0	—	4 (80)	—
Much improved	0	3 (75)	—	1 (20)	—
Minimally improved	1 (25)	0	—	0	—
No change	2 (50)	1 (25)	—	0	—
Minimally worse	1 (25)	0	—	0	—
Much worse	0	0	—	0	—
Very much worse	0	0	—	0	—

Adults with NT2					
	Placebo (*n* = 5)	Danavorexton 44 mg (*n* = 4)	*P* value 44 mg versus placebo	Danavorexton 112 mg (*n* = 5)	*P* value 112 mg versus placebo
**KSS** *					
Day 1	–0.4	–0.1	0.8089	–1.3	0.4311
Day 7	0.8	0.4	0.7407	–1.1	0.1155
**PVT** ^†^ ** (number of lapses)**					
Day 1	–3.4	–1.1	0.7089	–1.6	0.7281
Day 7	5.0	–4.6	0.1537	1.4	0.5180
**PVT** ^†^ ** (change in mean duration of the reciprocal 10% slowest reaction time [1/RT])**					
Day 1	–0.06	–0.22	0.6081	–0.08	0.9146
Day 7	–0.50	–0.12	0.2522	–0.15	0.2118
**PGI-C scale (reported 9 h after the start of infusion on day 7), *n* (%)**					
Very much improved	0	1 (25)	—	1 (25)	—
Much improved	0	2 (50)	—	3 (60)	—
Minimally improved	2 (40)	0	—	0	—
No change	3 (60)	1 (25)	—	1 (25)	—
Minimally worse	0	0	—	0	—
Much worse	0	0	—	0	—
Very much worse	0	0	—	0	—

^*^Mixed model for repeated measures analysis for change from a time-matched baseline period in KSS score.

^†^Mixed model for repeated measures analysis for change from a time-matched baseline period in PVT.

#### Cataplexy.

Prior to confinement at the clinical site in the MRD study, the mean numbers of cataplexy events during a time-matched 9-h baseline period from day −7 to day 1 were 2.3 (SD, 1.50), 5.8 (SD, 7.68), and 3.2 (SD, 2.59) for the placebo, danavorexton 11 mg, and danavorexton 44 mg groups, respectively. One individual in the danavorexton 11 mg cohort reported 17 cataplexy events during the baseline period, resulting in a higher baseline mean in that group.

Over the 7-d treatment period in the MRD study, the mean number of cataplexy episodes in individuals with NT1 during IV infusion decreased to zero in both danavorexton groups but not in the placebo group. Participants reported no cataplexy episodes during infusion of danavorexton 11 mg or 44 mg over the 7-d study period, compared with a mean number of cataplexy episodes of 1.8 during placebo infusion.

#### Additional PD outcomes assessed in the MRD study.

Functional alertness, measured via the Psychomotor Vigilance Task (PVT), improved in individuals with NT1 who received danavorexton, with a reduction seen in the mean number of lapses on day 1 and day 7. In individuals with NT2, no apparent trends in PVT outcomes were seen with danavorexton (Table 1). On the Patient Global Impression of Change (PGI-C), at 9 h after infusion initiation on day 7, most participants with NT1 who received danavorexton were either very much improved or much improved, whereas most participants in the placebo group reported no change on the PGI-C. A similar pattern was seen for participants with NT2 (Table 1).

#### Safety evaluations.

Across all study groups and cohorts in the SRD study (danavorexton 5–240 mg), treatment-emergent adverse events (TEAEs) were mild in severity, except for one moderate case of confirmed influenza reported in the danavorexton 7 mg group in the healthy adult cohort, which led to study drug discontinuation and was not considered related to study drug by the investigator. In the MRD study (danavorexton 11–180 mg), all TEAEs were mild in intensity, except for a moderate event of dysmenorrhea in the NT1 danavorexton 44 mg group, which was not considered related to study drug by the investigator. No serious TEAEs were reported during either study.

The most common TEAE among healthy adults in the SRD study was blood pressure (BP) increase (*SI Appendix*, Table S4). During administration of danavorexton 44.8 mg in individuals with NT1, systolic BP (SBP) and heart rate increases were reported in one participant; both occurred in the same participant, and both were judged to be related to the study drug by the investigator. An episode of hypnagogic hallucination also occurred several hours after the end of the infusion in one participant with NT1 who received danavorexton 44.8 mg. One participant with NT1 who received danavorexton 11.2 mg experienced several cataplexy episodes and sleep paralysis 14 h after the danavorexton infusion had been stopped, when no drug concentration was detectable in the plasma. Two TEAEs of PR interval prolongation were reported in the same healthy participant, once when receiving danavorexton 28 mg (from 180 to 235 ms) and again when receiving 112 mg (from 180 to 234 ms). The value returned to normal both times, ∼4 h after the start of infusion, during continued drug administration; no other electrocardiogram findings were reported.

The most common TEAE in the MRD study was pollakiuria, which occurred in four of five participants with NT1 in the danavorexton 44 mg group and in two of five participants with NT2 in the danavorexton 112 mg group (*SI Appendix*, Table S5). This TEAE was considered by investigators to be related to the study drug.

In healthy adults, at 9 h after the dose on day 7, placebo-adjusted changes in vital signs from time-matched baseline in the danavorexton 44, 112, and 180 mg groups, respectively, were: SBP, −0.1, −0.7, and 5.6 mmHg; diastolic BP (DBP), −1.3, −1.7, and 9.2 mmHg; and pulse rate, 4.6, 0.2, and 3.8 beats/min. In individuals with NT1, at 9 h after the dose on day 7, placebo-adjusted changes in vital signs in the danavorexton 11 mg and 44 mg groups, respectively, were: SBP, 1.3 and 0.0 mmHg; DBP, 8.2 and 5.9 mmHg; and pulse rate, 13.0 and 15.9 beats/min. In individuals with NT2, at 9 h after the dose on day 7, placebo-adjusted changes in vital signs in the danavorexton 44 mg and 112 mg groups, respectively, were: SBP, 14.0 and 9.4 mmHg; DBP, 12.6 and 9.3 mmHg; and pulse rate, 5.1 and 5.8 beats/min. No clinically significant changes from baseline laboratory values were reported in either study.

#### Pharmacokinetics.

After single IV infusions in healthy adults and individuals with NT1, mean plasma concentrations of danavorexton were proportional to dose over the dose range studied in both the SRD and the MRD studies (*SI Appendix*, Fig. S5). Plasma concentrations plateaued ∼3–4 h after the start of infusion. In healthy older adults, danavorexton plasma mean exposures were ∼18–27% higher than those in healthy younger adults, which is consistent with the observed 20% reduction in danavorexton clearance in older versus younger healthy participants. After completion of the infusion, danavorexton plasma concentrations declined rapidly. Across all groups in both studies, mean terminal disposition phase half-life ranged from ∼3.3 h to 5.1 h across all doses (*SI Appendix*, Tables S6 and S7). The mean CSF-to-plasma concentration ratio of danavorexton in healthy participants was estimated to be about 2.8% (*n* = 4; range 2.2–3.4%).

Following repeated dosing in the MRD study, the danavorexton concentration–time profiles in healthy adults, individuals with NT1, and individuals with NT2 were similar on days 1 and 7, and the accumulation ratios calculated from AUC_τ,ss_ (area under the plasma concentration–time curve during a dosing interval, at steady state) and AUC_τ_ (area under the plasma concentration–time curve during a dosing interval), and C_max,ss_ (maximum observed concentration, at steady state) and C_max_ (maximum observed concentration) were ∼1, indicating that once-daily, 9-h IV infusions for 7 d did not result in drug accumulation.

## Discussion

We report increased wakefulness after danavorexton treatment in a mouse model of NT1 that translated into significant improvement in excessive daytime sleepiness (EDS) in individuals with NT1, as measured using both objective (MWT, PVT) and subjective (ESS, KSS) endpoints. Individuals with NT2 also showed improved EDS with danavorexton treatment. In the narcolepsy mouse model, danavorexton was associated with decreased cataplexy-like episodes. Similarly, a potential signal was also seen in suppression of cataplexy in NT1, with no episodes occurring during treatment over 7 d. These results indicate that OX2Rs remain functional in individuals with NT1 despite long-standing loss of orexinergic neurons and, as such, OX2R-selective agonism has therapeutic potential in this disorder.

Orexin-deficient mice, compared with WT mice, exhibited hypersensitivity to danavorexton, a finding that is in line with previously reported research in which exogenous orexin A elicited stronger arousal effects in orexin-deficient mice compared with WT mice (21). Based on preclinical data, including this observed hypersensitivity, the danavorexton starting dose in the first NT1 cohort was lower than that in healthy volunteers. Correspondingly, individuals with NT1 displayed increased sensitivity to danavorexton compared with individuals with NT2, with similar improvements in mean sleep latency being observed with 5 mg for NT1 and 44 mg for NT2. Although mean sleep latency decreased over time following a low dose of 11 mg in individuals with NT1, sustained effectiveness was observed with 44 mg. More variable responses over time were observed in individuals with NT2 following doses of 44 mg or 112 mg. Differences measured via KSS between the treatment groups were also less clear in individuals with NT2 than in those with NT1, and there were no apparent trends in improvement in PVT outcomes in individuals with NT2. These findings support an apparent hypersensitivity to OX2R-selective agonism in NT1 and may also reflect the heterogeneity of the NT2 population. Differences between the NT1 and NT2 populations were observed at baseline, with adults with NT1 having slightly higher BMIs and slightly shorter sleep latencies than adults with NT2, which was consistent with previous reports (22, 23). Nonetheless, despite individuals with NT2 typically having normal orexin levels (24), OX2R-selective agonism appeared to exert a therapeutic effect.

All current narcolepsy pharmacotherapy relies on mechanisms of action unrelated to the underlying pathophysiology of NT1, namely the loss of orexinergic neurons in the lateral hypothalamus (4, 5, 25, 26). Common treatments include the use of amphetamine, methylphenidate, and the wakefulness-promoting agents modafinil and armodafinil for EDS (3, 27–29). In Europe and the United States, sodium oxybate is approved for the treatment of EDS and cataplexy (27, 28). Antidepressants, such as venlafaxine, are also used to manage cataplexy (28, 30, 31). Two recently introduced drugs offer additional treatment options for narcolepsy in Europe and the Unite States: the histamine H3 receptor antagonist/inverse agonist pitolisant for EDS and cataplexy, and the dopamine and norepinephrine reuptake blocker solriamfetol for EDS (15, 18, 32). No previous treatment has ever been shown to produce a complete MWT normalization of sleep latencies in individuals with NT1 (33–36), like that we show in the studies reported here, with sleep latencies reaching 40 min in almost all MWT trials during danavorexton administration.

Although loss-of-function mutations of OX1R and OX2R have not been reported in humans, the respective roles of OX1R and OX2R receptors in sleep regulation and narcolepsy can be inferred from animal studies and human genome-wide association studies. OX2R knockout mice show a clear narcolepsy phenotype that includes cataplexy. In contrast, OX1R knockout mice have minor or no sleep abnormalities and no cataplexy. However, dual receptor knockout mice show a more severe phenotype than that observed with the OX2R knockout mice, suggesting that OX1R also contributes to the pathophysiology of NT1 (37). Similarly, dogs with mutated OX2R exhibit narcolepsy with cataplexy, as do dogs lacking orexin neurons (38, 39). Finally, in humans, OX2R polymorphisms affect morningness–eveningness, napping, and reports of excessive daytime sleepiness, while OX1R polymorphisms have smaller but nominally significant effects on sleepiness and napping, based on numerous studies and data in the Sleep Disorder Knowledge Portal (https://sleep.hugeamp.org/) (40–43). In this study, consistent with the fact that a loss of OX1R signaling alone has no overt NT1-like phenotype, danavorexton, an OX2R-selective agonist, greatly improved the NT1 phenotype that results from a loss of orexin in mice and in human patients.

In both clinical studies, danavorexton was generally well tolerated, and the frequency of TEAEs was generally dose dependent in all study populations. BP increased with increasing danavorexton exposure, which was consistent with preclinical studies that have shown orexinergic stimulation to be associated with increased BP and heart rate (44, 45). Further assessment of OX2R-selective agonists will involve larger cohort sizes, longer duration of dosing, and use of ambulatory BP monitoring to provide a more accurate assessment of BP effects. Seven participants who received danavorexton experienced drug-related pollakiuria, which may be a result of the effects of danavorexton on the central nervous system. A study in rats identified OX2R expression in the pontine micturition center in the brainstem, which is responsible for bladder control (46). In addition, the parabrachial nucleus is innervated by orexinergic neurons and is thought to contribute to the functions of the pontine micturition center (24, 47, 48). Interestingly, this side effect also occurred at lower doses in individuals with NT1 than in those with NT2, again suggesting OX2R hypersensitivity. Of note, one participant who received danavorexton 11.2 mg experienced several cataplexy episodes and sleep paralysis 14 h after the infusion had been stopped, which may suggest a potential rebound effect.

Because of its poor oral bioavailability, danavorexton had to be administered via IV infusion. However, this feature enabled exact dosing control, making danavorexton ideal for exploring the potential of orexin agonists in the treatment of narcolepsy. The mean systemic exposures of danavorexton in healthy adults increased dose-proportionally, and systemic exposures were similar between healthy adults and individuals with NT1 or NT2. No accumulation of danavorexton was observed upon repeat dosing for 7 d, indicating that danavorexton has little or no potential for concentration-dependent or time-dependent cumulative pharmacological effects upon repeat dosing.

Strengths of the clinical studies include the crossover design used in the SRD study to increase the statistical power of the analysis. Because the design enabled evaluation of the effects of danavorexton within each person, the potential variance was lower than that of a parallel design with the same sample size. Additionally, several PD outcomes used in clinical trials of narcolepsy drugs were incorporated into both study designs, including validated objective and subjective measures that are standard for reporting the effects of therapeutic agents on EDS.

Limitations that should be considered include the inpatient setting of the clinical studies. Given the limited ambulatory activity and lack of external stimuli, few episodes of cataplexy were anticipated. Therefore, the potential for improvement of cataplexy with OX2R-selective agonists requires further investigation in an outpatient setting. Additionally, participant numbers were small, especially in the NT1 and NT2 groups, although this is expected in phase 1 studies. CSF orexin measurements were not obtained in these studies, so loss of orexin neuropeptides was presumed based on cataplexy and multiple sleep latency test results but not confirmed. Because of study design limitations, including the inpatient setting, IV administration, and limited dosing duration, an effect of danavorexton versus placebo could not be determined for sleep paralysis, hypnagogic/hypnopompic hallucinations, and disrupted nighttime sleep. BP was collected over several days only; longer dosing with ambulatory BP monitoring, the gold standard for collection of BP data, should be done in future studies. Therefore, available BP data should be interpreted with caution. The current trial was not designed to compare the effectiveness of danavorexton with that of previously approved narcolepsy medications.

In conclusion, we report promising results from preclinical mouse studies that translated into positive findings in human clinical studies in individuals with narcolepsy. Our findings strongly support the therapeutic potential of OX2R-selective agonists for the treatment of NT1 and NT2. Although IV administration is not optimal for use in an outpatient population with sleepiness, these results support the further evaluation of an orally available OX2R-selective agonist in individuals with sleep disorders characterized by EDS with or without reduced orexin levels.

## Materials and Methods

### Ethics.

Animal studies were approved by the Institutional Animal Care and Use Committee of Takeda Pharmaceutical Company Limited. Human studies (ClinicalTrials.gov identifiers: TAK-925–1001, NCT03332784; TAK-925–1003, NCT03748979) were approved by the Hakata Clinic Institutional Review Board and complied with Good Clinical Practice regulations and guidelines, and local regulations. Human studies were conducted in accordance with the ethical principles of the Declaration of Helsinki and met requirements and definitions of the International Council for Harmonization Harmonized Tripartite Guideline for Good Clinical Practice and all applicable local regulations. All participants gave informed consent.

### Orexin/ataxin-3 Mice and WT Littermate Studies.

We used orexin/ataxin-3 mice (20 wk old or older) on a C57BL/6J genetic background (University of Tsukuba) and WT littermates housed under 12-h light/dark cycles with food (CE-2, CLEA Japan Inc., Tokyo, Japan) and water ad libitum. Electroencephalographic and electromyographic recordings were performed in Takeda’s laboratories as described previously (19). Danavorexton (0.3, 1, 3, and 10 mg/kg subcutaneously [SC]), suspended in 0.5% methylcellulose saline, was administered at ZT12 (i.e., at the start of the 12-h dark cycle). SleepSign (Kissei Comtec Co., Ltd, Nagano, Japan) was used to evaluate sleep/wakefulness states in 4-s epochs. Locomotor activity was measured with an infrared activity sensor (Biotex, Kyoto, Japan). Murine cataplexy-like episodes were defined by a direct transition from wakefulness to REM sleep with no locomotion (49). For PK analyses, blood was collected 0.25, 0.5, 1, 2, 4, and 8 h after administration of danavorexton (0.3, 1, 3, and 10 mg/kg SC), and high-performance liquid chromatography–tandem mass spectrometry was used to quantify plasma concentrations of danavorexton (lower limit of detection 0.3 ng/mL).

Full methodological information and details of statistical analyses are provided in *SI Appendix* (*SI Appendix*, *SI Materials and Methods*).

### SRD and MRD Clinical Studies TAK-925–1001 and TAK-925–1003.

TAK-925–1001 was an SRD study of healthy volunteers and adults with NT1 (NCT03332784). TAK-925–1003 was an MRD study of healthy volunteers and adults with NT1 or NT2 (NCT03748979). Both studies were conducted at three sites in Japan, where danavorexton was administered as a continuous IV infusion over 9 h in saline. Both studies were designed to investigate: the safety and tolerability of danavorexton in healthy adults, older adults (SRD study only), and individuals with narcolepsy; the PK of single and multiple doses of danavorexton in healthy adults, older adults (SRD study only), and individuals with narcolepsy; and the effects of danavorexton in individuals with narcolepsy on sleep latency measured by the MWT, daytime sleepiness (measured by the Japanese version of the ESS) (50), and number of cataplexy episodes (NT1 only). Exploratory objectives included evaluating the effects of a single dose of danavorexton on sleepiness via the KSS (SRD study) and on alertness after multiple doses via the PVT (MRD study). Participants in the MRD study also reported the perceived efficacy of treatment via the PGI-C.

Redacted study protocols and statistical analysis plans are available at ClinicalTrials.gov (TAK-925–1001, https://clinicaltrials.gov/ProvidedDocs/84/NCT03332784/Prot_000.pdf and https://clinicaltrials.gov/ProvidedDocs/84/NCT03332784/SAP_001.pdf; TAK-925–1003, https://clinicaltrials.gov/ProvidedDocs/79/NCT03748979/Prot_001.pdf and https://clinicaltrials.gov/ProvidedDocs/79/NCT03748979/SAP_000.pdf).

Full methodological information and details of statistical analyses are provided in *SI Appendix*, *SI Materials and Methods*.

## Supplementary Material

Supplementary File

## Data Availability

The datasets, including the redacted study protocol, redacted statistical analysis plan, and individual participant data supporting the results reported in this article, will be available 3 months from initial request to researchers who provide a methodologically sound proposal. The data will be provided after its deidentification, in compliance with applicable privacy laws, data protection, and requirements for consent and anonymization.

## Data, Materials, and Software Availability

The datasets, including the redacted study protocol, redacted statistical analysis plan, and individual participant data supporting the results reported in this article, will be available 3 months from initial request to researchers who provide a methodologically sound proposal. The data will be provided after its deidentification, in compliance with applicable privacy laws, data protection, and requirements for consent and anonymization.
