# Supplementary material for: Orexin 2 receptor–selective agonist danavorexton improves narcolepsy phenotype in a mouse model and in human patients
Source: Proc Natl Acad Sci U S A. 2022 Aug 22;119(35):e2207531119. doi: 10.1073/pnas.2207531119 (PMC9436334; doi:10.1073/pnas.2207531119)
Supplement: Supplementary File [file pnas.2207531119.sapp.pdf]

Supplementary Information for

Orexin 2 receptor-selective agonist danavorexton improves narcolepsy phenotype in a mouse model and in human patients

Rebecca Evans, Haruhide Kimura, Robert Alexander, Ceri H. Davies, Hélène Faessel, Deborah S. Hartman, Takashi Ishikawa, Emiliangelo Ratti, Kohei Shimizu, Motohisa Suzuki, Shinichiro Tanaka, Hiroshi Yukitake, Yves Dauvilliers, Emmanuel Mignot*

*Corresponding author: Emmanuel Mignot

Email: mignot@stanford.edu

This PDF file includes:

Supplementary Materials and Methods

Figures S1 to S5

Tables S1 to S9

SI References

Supplementary Information Text

Preclinical study methods

Ethics

Animal studies were approved by the Institutional Animal Care and Use Committee of Takeda Pharmaceutical Company Limited.

Orexin/ataxin-3 mice and their WT littermates

Orexin/ataxin-3 mice on a C57BL/6J genetic background were provided by the University of Tsukuba. Orexin/ataxin-3 mice and their WT littermate mice were bred in Takeda's laboratories. Twenty-week-old or older male mice were used for EEG, EMG, and locomotor activity monitoring. All mice were housed under laboratory conditions (12-hour light/dark cycles) with food (CE-2, CLEA Japan Inc., Tokyo, Japan) and water available ad libitum.

Chemicals, surgical procedures, and data acquisition

Danavorexton (0.3, 1, 3, and 10 mg/kg subcutaneously [SC]), suspended in 0.5% methylcellulose saline, was administered to orexin/ataxin-3 mice at ZT12 (i.e., at the start of the 12-hour dark cycle) in a volume of 10 mL/kg of body weight. EEG, EMG, and locomotor activity were recorded for scoring of sleep and activity. Implantation of EEG/EMG electrodes and EEG/EMG recordings were performed as described previously (1).

SleepSign (Kissei Comtec Co., Ltd, Nagano, Japan) was used to classify sleep/wakefulness states in 4-second epochs as wakefulness, NREM sleep, or REM sleep according to standard criteria. This software was also used to calculate total time, duration, and number of each sleep/wakefulness state. Locomotor activity was measured by an infrared activity sensor (Biotex, Kyoto, Japan).

Data analyses for sleep/wakefulness fragmentation and cataplexy-like episodes in orexin/ataxin-3 mice and WT mice

Analyses of sleep/wakefulness were carried out on data continuously collected during the first hour after administration (1). Murine cataplexy is associated with direct transitions from

wakefulness to REM sleep and is accompanied by sudden behavioral arrest. In this study, murine cataplexy-like episodes were defined by the direct transition from wakefulness to REM sleep with no locomotion (2). EEG and EMG recordings, together with locomotor measurement by infrared activity sensors, were used to count the number of cataplexy-like episodes based on the following criteria: (1) abrupt episode of EMG atonia lasting ≥20 seconds; (2) behavioral immobility during the episode (assessed by locomotion); (3) predominance of theta activity during the episode; and (4) ≥40 seconds of wakefulness preceding the episode. In a separate group of orexin/ataxin-3 mice, immobility during the cataplexy-like episodes was further confirmed using video recording. Palatable food, such as chocolate, has been known to increase the number of cataplexy-like episodes in mice with orexin deficiency during the active phase (3, 4). Danavorexton (0.3 mg/kg and 1 mg/kg SC) was administered to orexin/ataxin-3 mice at ZT12, then milk chocolate (Hershey's Kisses milk chocolate, Hershey, PA, USA) was given to mice, and EEG, EMG, and locomotor activity were recorded. The number of cataplexy-like episodes during the 3-hour period after danavorexton administration was counted.

Blood samples for PK analyses were collected at various time points (0.25, 0.5, 1, 2, 4, and 8 hours) after administration of danavorexton (0.3, 1, 3, and 10 mg/kg SC) in C57BL/6J mice (CLEA Japan Inc., Tokyo, Japan). Plasma concentration of danavorexton was quantified with high-performance liquid chromatography–tandem mass spectrometry (HPLC–MS/MS). The lower limit of quantitation of danavorexton was 0.3 ng/mL for plasma.

Effects of danavorexton on wakefulness during sleep/inactive phase in WT mice and orexin/ataxin-3 mice

Evaluation of drug efficacy on sleep/wakefulness states in mice was conducted using a pre-test–post-test design. Danavorexton (0.1, 0.3, 1, or 3 mg/kg SC) was administered to WT mice or orexin/ataxin-3 mice at ZT5 (note: start of the lights-on period is ZT0), and then EEG/EMG were recorded.

Effects of repeated administration of danavorexton on sleep/wakefulness states and cataplexy-like episodes in orexin/ataxin-3 mice

Orexin/ataxin-3 mice were grouped into three cohorts: control group, vehicle–danavorexton group, and danavorexton–danavorexton group. In the control group, vehicle (0.5% methylcellulose saline) was administered SC to mice twice daily at ZT12 and ZT15 for 13 days. The following day (day 14), vehicle was administered at ZT12, and EEG/EMG were recorded from ZT12. On day 15, vehicle was administered at ZT12, then milk chocolate was placed in the cage, followed by EEG, EMG, and locomotor activity recordings. In the vehicle–danavorexton group, vehicle was administered SC to mice at ZT12 and ZT15 for 13 days. On day 14, danavorexton (3 mg/kg SC) was administered at ZT12, and EEG/EMG were recorded from ZT12. On day 15, danavorexton (3 mg/kg SC) was administered at ZT12, then milk chocolate was placed in the cage, followed by EEG, EMG, and locomotor activity recordings. In the danavorexton–danavorexton group, danavorexton (3 mg/kg SC) was administered to mice at ZT12 and ZT15 for 13 days. On day 14, danavorexton (3 mg/kg SC) was administered at ZT12, and EEG/EMG were recorded from ZT12. On day 15, danavorexton (3 mg/kg SC) was administered at ZT12, then milk chocolate was placed in the cage, followed by EEG, EMG, and locomotor activity recordings. The time spent in wakefulness, NREM sleep, and REM sleep (day 14), the number and duration of wakefulness episodes (day 14), and the number of cataplexy-like episodes (day 15) were calculated.

Statistics

Data are presented as mean ± standard error of the mean, or mean ± SD. Statistical analysis was performed using EXSUS (CAC EXICARE Corporation, Tokyo, Japan). To compare two unpaired groups (i.e., orexin/ataxin-3 mice versus WT mice), data were analyzed with a two-tailed Student's *t*-test (for data with homoscedasticity) or a two-tailed Aspin–Welch test (for data with heteroscedasticity). Evaluation of danavorexton efficacy on sleep/wakefulness states and cataplexy-like episodes in mice was conducted by comparing pre-test and post-test effects. Thus, pairwise differences between groups (i.e., vehicle versus danavorexton) were evaluated using a

two-tailed paired *t*-test. Multiplicity of testing was controlled using a closed testing procedure from the high dose side. To compare time-elapsed data (i.e., time course of wakefulness time), data were analyzed with repeated measures analysis of variance followed by a post hoc two-tailed paired *t*-test with closed testing procedure from the first time point after drug administration. In the repeated dosing analyses, comparisons between groups were conducted using two-tailed Tukey's test. For all analyses, a *P* value of ≤0.05 was considered significant.

SRD and MRD clinical studies TAK-925-1001 and TAK-925-1003

Redacted study protocols and statistical analysis plans are available at ClinicalTrials.gov (TAK-925-1001: https://clinicaltrials.gov/ProvidedDocs/84/NCT03332784/Prot_000.pdf and https://clinicaltrials.gov/ProvidedDocs/84/NCT03332784/SAP_001.pdf; TAK-925-1003: https://clinicaltrials.gov/ProvidedDocs/79/NCT03748979/Prot_001.pdf and https://clinicaltrials.gov/ProvidedDocs/79/NCT03748979/SAP_000.pdf).

Ethics

The SRD and MRD studies were approved by the Hakata Clinic Institutional Review Board. These complied with Good Clinical Practice (GCP) regulations and guidelines, and local regulations (5). Both studies were registered on ClinicalTrials.gov (TAK-925-1001, NCT03332784; TAK-925-1003, NCT03748979). Studies were conducted in accordance with ethical principles that have their origin in the Declaration of Helsinki, the requirements and definitions of the International Council for Harmonisation (ICH) Harmonised Tripartite Guideline for GCP, and all applicable local regulations. All participants signed and dated informed consent forms before any protocol-specific screening procedures took place.

Study objectives

The primary objectives were: to investigate the safety and tolerability of danavorexton in healthy adults and in individuals with NT1 (both studies) and in individuals with NT2 (MRD study only); and to investigate the PK of a single dose of danavorexton in healthy adults, elderly participants, and individuals with NT1 (SRD study only).

The secondary objectives were: to investigate the PK of multiple doses of danavorexton in healthy adults and in individuals with NT1 and NT2 (the MRD study only); and to investigate the PD of danavorexton after single IV administration in individuals with NT1 (SRD study) and multiple IV administration in individuals with NT1 and NT2 (MRD study), primarily by evaluating sleep latency on MWT, daytime sleepiness (as measured by the Japanese version of the ESS [JESS]) (6), and number of cataplexy episodes (NT1 only). Exploratory objectives included evaluating the PD effects of danavorexton when administered as a single dose using the KSS (SRD study), and after multiple doses using the PVT and PGI-C (MRD study).

Study design and schedule of procedures

Both studies were conducted at three sites in Japan. Danavorexton was administered as a continuous IV infusion over 9 hours in saline. Rationale for dose selection for the first-in-human SRD study followed US Food and Drug Administration guidance on human equivalent dose calculation, based on allometric scaling and no observed adverse effect levels determination in the most sensitive species (7). In addition, pharmacology studies conducted in mice were taken into consideration. Observed differences in sensitivity between WT and orexin/ataxin-3 mice led to the targeted plasma concentrations in the first NT1 cohorts. The first dose-level cohort (danavorexton 7 mg) was designed to obtain safety and tolerability information following a single dose of danavorexton administered via IV infusion, as well as to obtain the information on PK parameters that would determine the infusion rate and dose levels in subsequent cohorts. Subsequent doses were determined depending on the available safety, tolerability, and PK data of previous doses. In the MRD study, dose escalation in all parts was determined based on the available safety, tolerability, PK, and PD data from previous cohorts and from the SRD study. In individuals with NT1, a starting dose of 11 mg was considered to be appropriate to examine safety, PK, and PD when dosed daily for 7 days. An effective dose level for individuals with NT2 was considered to be similar to that for healthy volunteers, rather than the dose for those with NT1.

The SRD study part 1 was a randomized, placebo-controlled study, which first evaluated cohorts receiving a single IV dose of danavorexton 7–134.4 mg compared with placebo. Block randomization was used to ensure that six participants received danavorexton and two participants received placebo in each dosing period. Two additional, subsequent parallel cohorts of eight participants were randomized 6:2 to danavorexton and placebo, and they received higher doses of 180 mg and 240 mg each. One parallel cohort of healthy elderly participants (n = 8, randomized 6:2 to danavorexton 112 mg and placebo) was evaluated. One open-label cohort of healthy participants receiving danavorexton 112 mg (n = 4) had CSF collection for PK analysis.

The SRD study part 2 consisted of three NT1 cohorts, each conducted as a randomized, double-blind (except for a sponsor unblinded team), placebo-controlled, two-period, crossover study to assess the effect of danavorexton on maintaining wakefulness. The three cohorts were dosed as follows: danavorexton 5 mg and placebo (n = 6); danavorexton 11.2 mg and placebo (n = 4); and danavorexton 44.8 mg and placebo (n = 4).

All three parts of the MRD study were randomized, double-blind, placebo-controlled studies to assess the safety, tolerability, and PK of danavorexton administered via IV infusion in healthy adults (part A), individuals with NT1 (part B), and individuals with NT2 (part C). Part B and part C also included assessments of PD effects in NT1 and NT2. In all parts of the MRD study, danavorexton or placebo was administered via IV infusion over 9 hours once daily for 7 days.

The MRD study part A was planned as two cohorts (A1 and A2; danavorexton 44 mg and 112 mg, respectively) plus one additional cohort (A3; danavorexton 180 mg). Each cohort consisted of eight healthy adults randomized to danavorexton or placebo in a 6:2 ratio in a double-blinded fashion.

The MRD study part B was planned as two cohorts (B1 and B2; danavorexton 11 mg and 44 mg, respectively). Each cohort consisted of six individuals with NT1 who were randomized to danavorexton or placebo in a 4:2 ratio in a double-blinded fashion. In part B, an exploratory PD assessment was conducted to evaluate the potential efficacy of danavorexton using MWT, KSS, cataplexy frequency assessment, PVT, and PGI-C.

The MRD study part C was planned as two cohorts (C1 and C2; danavorexton 44 mg and 112 mg, respectively). Each cohort consisted of six individuals with NT2 who were randomized to danavorexton or placebo in a 4:2 ratio in a double-blinded fashion.

Participants underwent NPSG on day −2 and baseline MWT sessions four times on day −1 (at approximately 10:00, 12:00, 14:00, and 16:00). Study drug dosing via IV infusion was started at approximately 08:00. MWT was conducted on day 1 at approximately 10:00, 12:00, 14:00, and 16:00. Participants were allowed to take a nap on the days with no MWT assessment. On day 7, the participants underwent MWT at the same hours as on day −1. Cataplexy was recorded using a sleep diary.

Study populations

The SRD and MRD studies both enrolled healthy adults and individuals with NT1. The SRD study also enrolled healthy elderly participants, while the MRD study enrolled individuals with NT2. In the SRD study part 1 and the MRD study part A, eligible healthy adults were 20–55 years of age (inclusive), had a BMI of ≥18.5 kg/m^2 but ≤30.0 kg/m^2, had a body weight of ≥50 kg, and were normotensive at screening (SBP <140 mmHg and DBP <90 mmHg) with no history of hypertension or use of antihypertensive medication. In the SRD study part 1, eligible healthy elderly participants were 65–80 years of age (inclusive), had a BMI of ≥18.5 kg/m^2 but ≤30.0 kg/m^2, had a body weight of ≥40 kg and were normotensive at screening (SBP <140 mmHg and DBP <90 mmHg) with no history of hypertension or use of antihypertensive medication.

Participants with any significant comorbid medical disorders were excluded. All stimulants and anti-cataplectic medications were stopped prior to the baseline visit for ≥1 week (tables S8 and S9; none of the participants had used sodium oxybate).

In the SRD study part 2 and the MRD study part B, eligible participants had a diagnosis of NT1 as defined by the International Classification of Sleep Disorders-Third Edition, an ESS score of ≥10, an average (of four sessions) baseline MWT sleep latency of ≤20 minutes, and no sleep latency of ≥30 minutes in any single session, and they had tested positive for HLA-DQB1*06:02. They were 18–80 years of age (inclusive), had a body weight of ≥40 kg, and had an SBP <140 mmHg

and DBP <90 mmHg. In the MRD study, participants with NT1 had to have experienced at least three episodes of cataplexy per week during the screening period.

In the MRD study, inclusion criteria for participants with NT2 were the same as for participants with NT1, except that participants had a diagnosis of NT2 as defined by the ICSD-3 and there was no cataplexy criterion.

PK evaluation

Plasma (SRD and MRD) and CSF (SRD only) concentrations of danavorexton were measured using validated HPLC–MS/MS methods. In the SRD study part 1, blood samples for PK analyses were collected from healthy participants before infusion, at 0.5, 1, 1.5, 2, 3, 4, 6, 8, and 9 hours after the start of infusion, and at 0.17, 0.33, 0.5, 0.75, 1, 1.5, 2, 3, 4, 6, 10, and 15 hours after the end of infusion. In the SRD study part 2, blood samples from individuals with NT1 were collected before infusion, at 1, 2, 4, 6, and 9 hours after the start of infusion, at 0.17, 0.5, 1, 2, and 15 hours after the end of infusion, at bedtime, and upon awakening. In the SRD study part 1, CSF samples (2.5 mL each) were taken 6 hours after the start of infusion for the CSF cohort only (healthy participants, n = 4, SRD study part 1).

In the MRD study part A, blood samples for PK analysis were taken before the start of infusion, 0.5, 1, 1.5, 2, 4, 6, 8, and 9 hours after start of infusion, and 0.17, 0.33, 0.5, 0.75, 1, 1.5, 2, 3, 6, 10, and 15 hours after end of infusion on days 1 and 7. In the MRD study parts B and C, blood samples were taken before the start of infusion, 1, 2, 4, 6, and 9 hours after start of infusion, and 0.17, 0.5, 2, 6, 10, and 15 hours after end of infusion on days 1 and 7. For all parts of the MRD study, blood samples were also taken before the start of infusion and 9 hours after the start of infusion on days 5 and 6.

The lower limit of quantitation of danavorexton was 0.2 ng/mL for plasma and 0.1 ng/mL for CSF.

PD assessments (SRD study part 2 and MRD study parts B and C)

The MWT is a validated objective measure of the time taken for a person to fall asleep under soporific conditions (semi-reclined position and trying to stay awake, lights on) as determined

using EEG. The MWT has been routinely used in clinical trials of drugs approved to treat EDS in narcolepsy (8, 9). Four 40-minute sessions of the MWT were performed at approximately 10:00, 12:00, 14:00, and 16:00, and participants were required to stay awake during intervals between sessions. In the SRD study, the MWT was performed on day 1 only. In the MRD study, the MWT was performed on day −1, day 1, and day 7.

The KSS is a Likert-type, nine-level, self-report scale that measures the subjective level of sleepiness at a specific time point during the day. Participants indicate which level best reflects their sleepiness experienced in the past 10 minutes (10). The KSS was rated at baseline and hourly until 11 hours after infusion start. In the SRD study, the KSS was performed on day −1 and day 1. In the MRD study, the KSS was performed on day −1, day 1, and day 7.

The ESS measure of EDS is a subjective, self-recorded rating scale of the tendency to fall asleep in eight defined situations of daily life. A four-point scale (scored at 0–3) is used to answer each of the eight questions to give a total in the range 0–24. Higher scores indicate stronger subjective daytime sleepiness, and scores <10 are considered to be normal. In studies TAK-925-1001 and TAK-925-1003, the JESS was used to evaluate subjective sleepiness, and participants were asked to consider the past 7 days when completing the survey (6). In the SRD study, the ESS was only used as a baseline measure. In the MRD study, the ESS was assessed on day −1 and day 7.

The PVT is a simple reaction performance task that measures behavioral alertness and sustained attention by recording reaction times to visual stimuli. In study TAK-925-1003, a 10-minute version of PVT was used; an inter-stimulus interval was set at between 2 seconds and 10 seconds. In the MRD study only, the PVT was assessed on day 1, day −1, and day 7.

The PGI-C is a seven-point self-report measure of the perceived efficacy of treatment. Patients rate their perceived change from ‘very much improved’ to ‘very much worse.’ In the MRD study only, the PGI-C was assessed on day 7.

Assessment of cataplexy episodes (the MRD study part B only) was conducted in individuals with NT1 based on sleep diary and self-reported questionnaires, and time of event(s) was captured.

Safety assessments

For both studies, a TEAE was defined as an adverse event (AE) that occurred on or after the start of study drug administration. A related TEAE was defined as an AE that followed a reasonable temporal sequence from administration of a study drug, or for which a causal relationship was at least a reasonable possibility. Safety measures included: reported AEs; vital signs, including pulse rate, and BP (BP was checked with the participant lying in a bed with the head of the bed inclined at 30 degrees); body weight; 12-lead and Holter ECG; and clinical laboratory tests (hematology, chemistry, urinalysis).

Statistical analysis

For each study, three analysis sets were used: the safety set (all participants who received at least one dose of study drug); the PK set (all participants who received at least one dose of study drug and provided sufficient PK measurements to estimate at least one PK parameter); and the PD set (all participants who received at least one dose of study drug). Plasma and CSF concentrations of danavorexton were summarized for each scheduled sampling time using descriptive statistics. Plasma and CSF PK parameters of danavorexton were summarized using descriptive statistics. In both studies, PD outcomes were described using descriptive statistics and, when appropriate in the MRD study, the effect of danavorexton was evaluated with a linear mixed effects model. The model included treatment (each dose level of danavorexton and placebo), day (as a categorical variable), the treatment-by-day as fixed effects, baseline average sleep latency in the MWT as a covariate, and participant as a random effect. TEAEs were coded using the Medical Dictionary for Regulatory Activities.

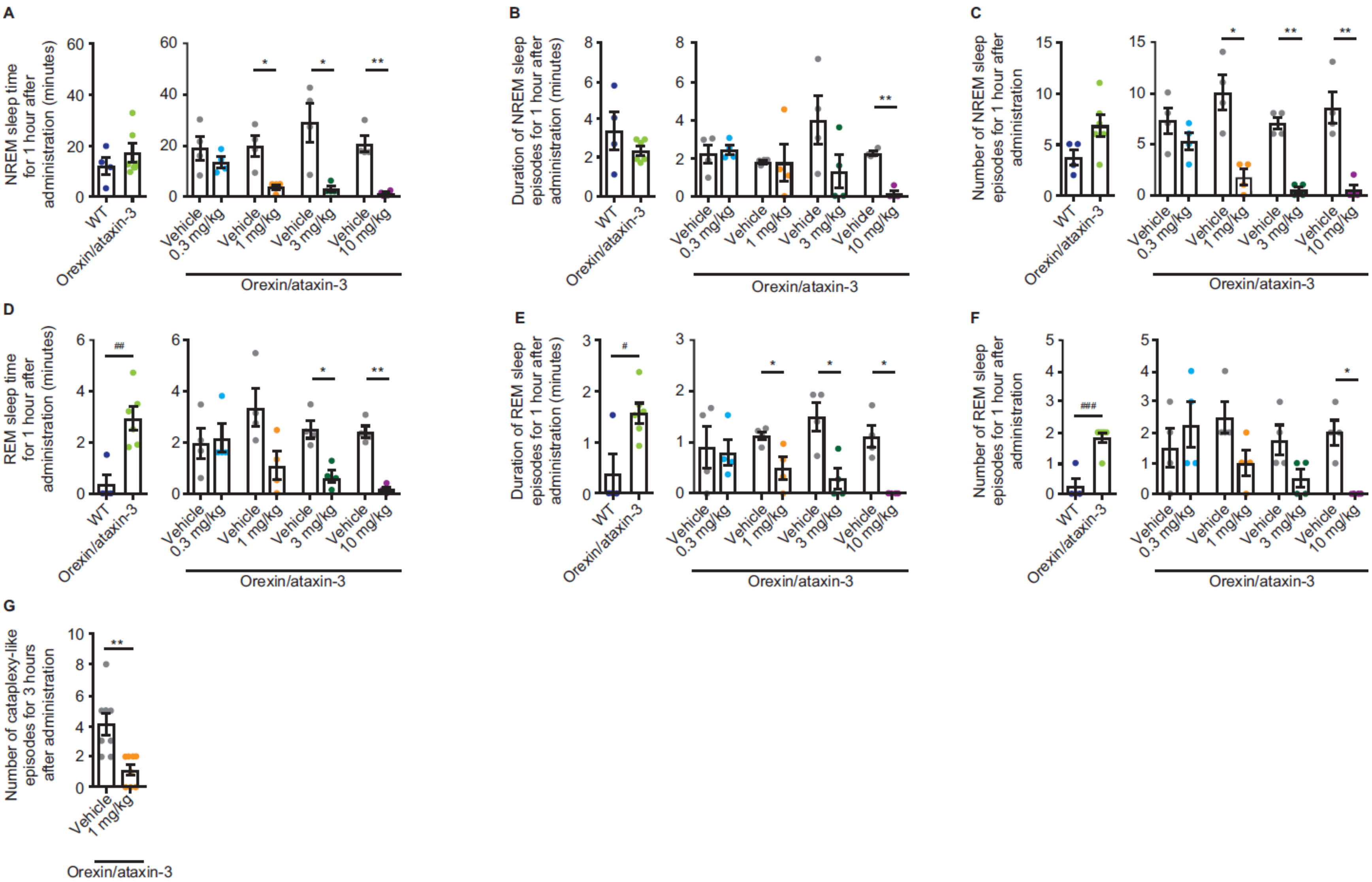

A
NREM sleep time for 1 hour after administration (minutes)
WT
Orexin/ataxin-3
Vehicle
0.3 mg/kg
Vehicle
1 mg/kg
Vehicle
3 mg/kg
Vehicle
10 mg/kg
Orexin/ataxin-3
B
Duration of NREM sleep episodes for 1 hour after administration (minutes)
C
Number of NREM sleep episodes for 1 hour after administration
D
REM sleep time for 1 hour after administration (minutes)
E
Duration of REM sleep episodes for 1 hour after administration (minutes)
F
Number of REM sleep episodes for 1 hour after administration
G
Number of cataplexy-like episodes for 3 hours after administration
Vehicle
1 mg/kg
Orexin/ataxin-3

Fig. S1. Danavorexton effects on sleep/wakefulness fragmentation and cataplexy-like episodes in orexin/ataxin-3 mice: NREM and REM sleep analysis. A,B,C, Effect of danavorexton single doses (0.3, 1, 3 and 10 mg/kg SC) on total NREM sleep time (**A**), duration of NREM sleep episodes (**B**) and number of NREM sleep episodes (**C**) in orexin/ataxin-3 mice during active phase. **D,E,F,** Effect of danavorexton single doses (0.3, 1, 3 and 10 mg/kg SC) on total REM sleep time (**D**), duration of REM sleep episodes (**E**) and number of REM sleep episodes (**F**) in orexin/ataxin-3 mice during active phase. **G**, Effect of danavorexton single doses (1 mg/kg SC) on cataplexy-like episodes in orexin/ataxin-3 mice during active phase. Behavioral immobility during the cataplexy-like episodes detected by EEG/EMG/locomotion measurement in mice was confirmed by video data. #$P \leq 0.05$, ##$P \leq 0.01$, ###$P \leq 0.001$ (two-tailed Student *t*-test; WT, $n = 4$; orexin/ataxin-3, $n = 6$), *$P \leq 0.05$, **$P \leq 0.01$ (two-tailed paired *t*-test with closed testing procedure from the high dose side; **A–F**, $n = 4$; **G**, $n = 8$). Mean ± SEM. EEG, electroencephalography; EMG, electromyography; NREM, non-rapid eye movement; REM, rapid eye movement; SC, subcutaneously; SEM, standard error of the mean; WT, wild type.

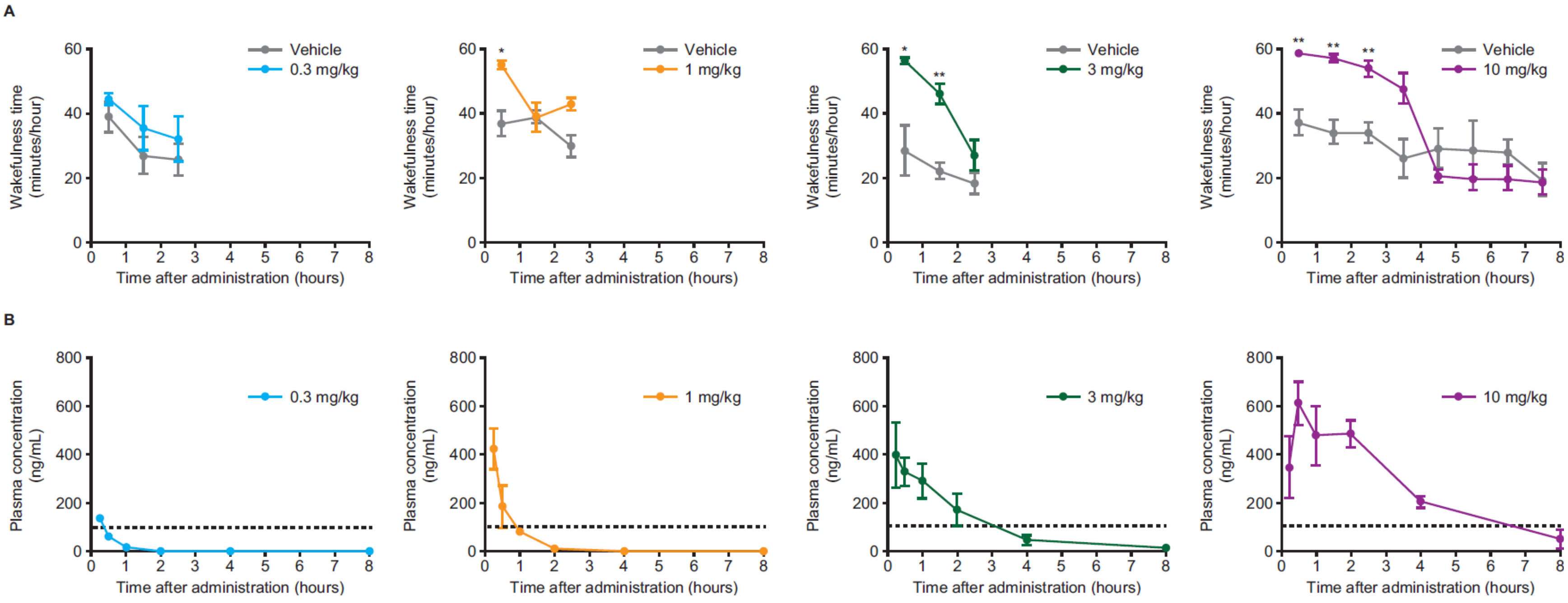

Fig. S2. Time course of wakefulness in orexin/ataxin-3 mice and plasma concentration after administration of danavorexton in WT mice. **A**, Time course of wakefulness time during 3 hours (0.3, 1 and 3 mg/kg) or 8 hours (10 mg/kg) in vehicle- or danavorexton-treated orexin/ataxin-3 mice. *$P \leq 0.05$, **$P \leq 0.01$ versus vehicle-treated mice at each time point (using repeated measures ANOVA followed by a *post hoc* two-tailed paired *t*-test with closed testing procedure from the first time point after drug administration). Mean ± SEM, $n = 4$. **B**, Plasma concentration after subcutaneous administration of danavorexton in C57BL/6J mice. Dotted line indicates the threshold concentration for wakefulness (approximately 100 ng/mL). Mean ± SD, $n = 3$. ANOVA, analysis of variance; SD, standard deviation; SEM, standard error of the mean; WT, wild type.

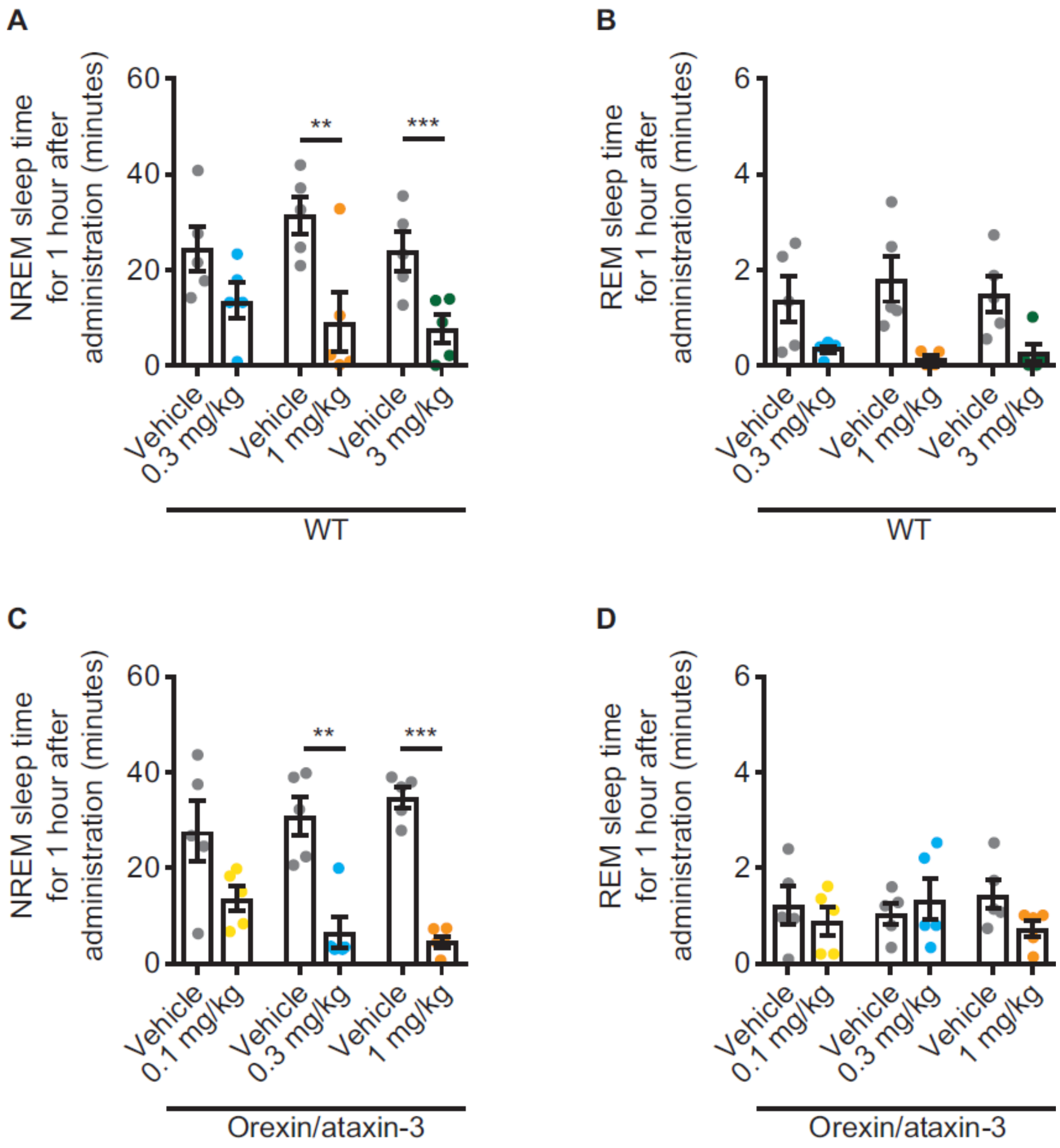

Fig. S3. Danavorexton sensitivity analyses in orexin/ataxin-3 mice and WT littermates: NREM and REM sleep analysis. A,B, Effects of danavorexton on NREM sleep time (**A**) and REM sleep time (**B**) in WT mice (n = 5). **C,D**, Effects of danavorexton on NREM sleep time (**C**) and REM sleep time (**D**) in orexin/ataxin-3 mice (n = 5). Mean ± SEM. **$P \leq 0.01$, ***$P \leq 0.001$ (two-tailed paired t-test with closed testing procedure from the high dose side). NREM, non-rapid eye movement; REM, rapid eye movement; SEM, standard error of the mean; WT, wild type.

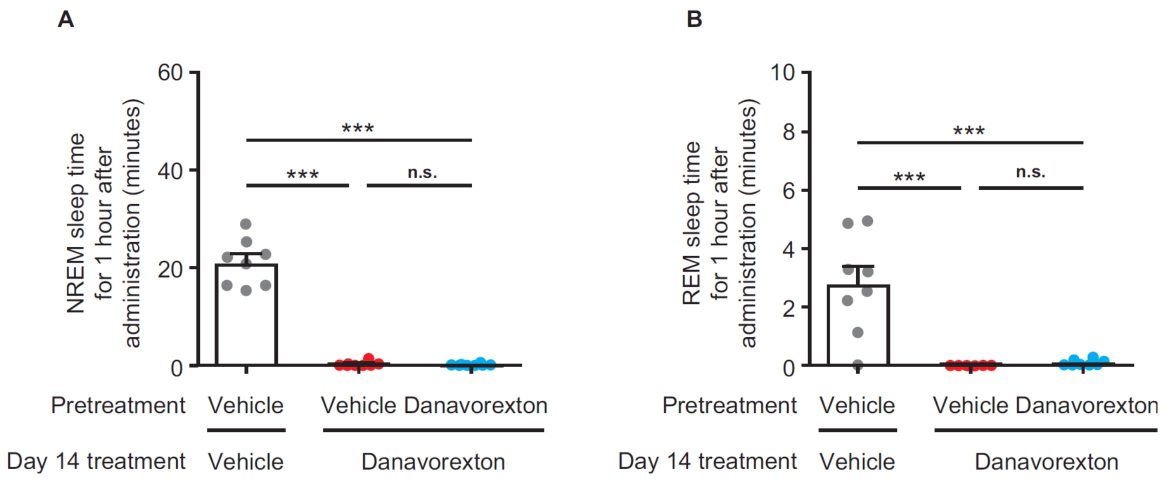

Fig. S4. Effects of danavorexton chronic dosing in orexin/ataxin-3 mice: NREM and REM sleep analysis. Danavorexton decreased NREM sleep time and REM sleep time after single and repeated administration in orexin/ataxin-3 mice during the active phase. **A**,**B**, Effects of single and repeated administration of danavorexton on NREM sleep time (**A**) and REM sleep time (**B**) in orexin/ataxin-3 mice (n = 8). Mean ± SEM. ***$P \leq 0.001$ (two-tailed Tukey's test). NREM, non-rapid eye movement; n.s., not significant; REM, rapid eye movement; SEM, standard error of the mean.

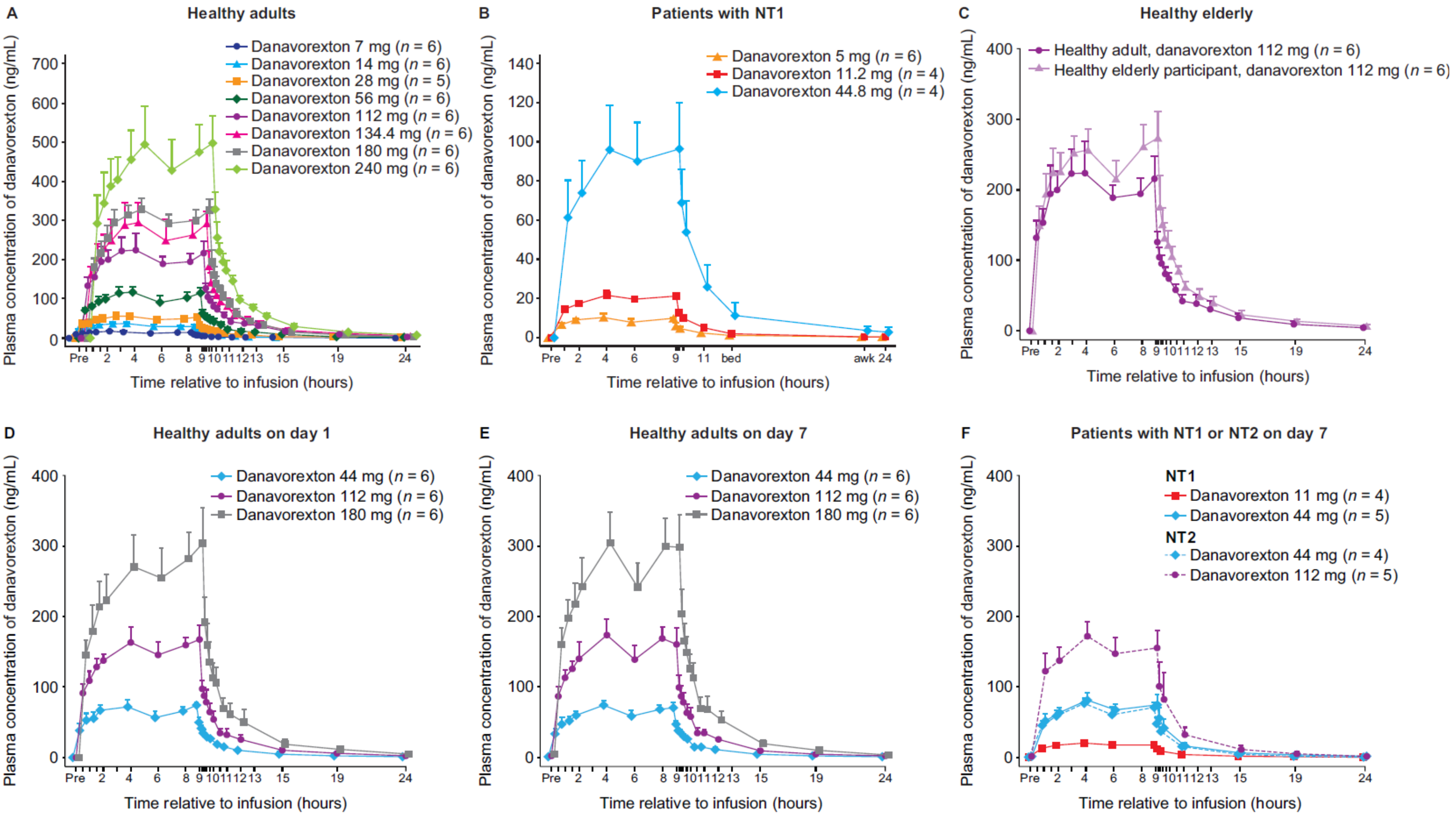
A
Healthy adults
Danavorexton 7 mg (n = 6)
Danavorexton 14 mg (n = 6)
Danavorexton 28 mg (n = 5)
Danavorexton 56 mg (n = 6)
Danavorexton 112 mg (n = 6)
Danavorexton 134.4 mg (n = 6)
Danavorexton 180 mg (n = 6)
Danavorexton 240 mg (n = 6)
Plasma concentration of danavorexton (ng/mL)
Time relative to infusion (hours)
B
Patients with NT1
Danavorexton 5 mg (n = 6)
Danavorexton 11.2 mg (n = 4)
Danavorexton 44.8 mg (n = 4)
Plasma concentration of danavorexton (ng/mL)
Time relative to infusion (hours)
Pre 2 4 6 9 11 bed awk 24
C
Healthy elderly
Healthy adult, danavorexton 112 mg (n = 6)
Healthy elderly participant, danavorexton 112 mg (n = 6)
Plasma concentration of danavorexton (ng/mL)
Time relative to infusion (hours)
D
Healthy adults on day 1
Danavorexton 44 mg (n = 6)
Danavorexton 112 mg (n = 6)
Danavorexton 180 mg (n = 6)
Plasma concentration of danavorexton (ng/mL)
Time relative to infusion (hours)
E
Healthy adults on day 7
Danavorexton 44 mg (n = 6)
Danavorexton 112 mg (n = 6)
Danavorexton 180 mg (n = 6)
Plasma concentration of danavorexton (ng/mL)
Time relative to infusion (hours)
F
Patients with NT1 or NT2 on day 7
NT1
Danavorexton 11 mg (n = 4)
Danavorexton 44 mg (n = 5)
NT2
Danavorexton 44 mg (n = 4)
Danavorexton 112 mg (n = 5)
Plasma concentration of danavorexton (ng/mL)
Time relative to infusion (hours)

Fig. S5. Plasma concentrations of danavorexton during 9-hour infusion. **A**,**B**,**C**,**D**,**E**,**F**, Mean plasma pharmacokinetic profiles of danavorexton during a single 9-hour infusion in the SRD study TAK-925-1001 for healthy adults (**A**), individuals with NT1 (**B**) and healthy elderly participants (**C**), and in the MRD study TAK-925-1003 for healthy volunteers on day 1 (**D**) and day 7 (**E**), and in participants with NT1 or NT2 (**F**) on day 7. Data points and error bars represent mean and standard deviation values, respectively (mg/mL). Note that the axes vary to reflect the different dose levels administered. awk, awake; MRD, multiple rising dose; NT1, narcolepsy type 1; NT2, narcolepsy type 2; SRD, single rising dose

Table S1. Demographics and baseline characteristics of healthy volunteers in the single-rising-dose study

	Healthy adults										Healthy elderly	
	Cohorts 1, 2, S1 and S2									CSF		
	Placebo (*n* = 15)	danavorexton 7 mg (*n* = 6)	danavorexton 14 mg (*n* = 6)	danavorexton 28 mg (*n* = 5)	danavorexton 56 mg (*n* = 6)	danavorexton 112 mg (*n* = 6)	danavorexton 134.4 mg (*n* = 6)	danavorexton 180 mg (*n* = 6)	danavorexton 240 mg (*n* = 6)	danavorexton 112 mg (*n* = 4)	Placebo (*n* = 2)	danavorexton 112 mg (*n* = 6)
Age, years												
Median (min–max)	27.0 (20–38)	24.5 (20–33)	24.5 (21–35)	29.0 (25–33)	29.0 (21–38)	26.0 (20–33)	29.0 (22–38)	22.5 (20–35)	25.0 (22–40)	21.0 (21–22)	72.5 (70–75)	66.5 (65–73)
Sex, *n* (%)												
Male	15 (100.0)	6 (100.0)	6 (100.0)	5 (100.0)	6 (100.0)	6 (100.0)	6 (100.0)	6 (100.0)	6 (100.0)	4 (100.0)	1 (50.0)	3 (50.0)
Female	0 (0.0)	0 (0.0)	0 (0.0)	0 (0.0)	0 (0.0)	0 (0.0)	0 (0.0)	0 (0.0)	0 (0.0)	0 (0.0)	1 (50.0)	3 (50.0)
BMI, kg/m^2												
Median (min–max)	22.00 (18.8–25.5)	22.30 (18.8–24.0)	21.35 (19.7–25.3)	22.20 (20.5–24.0)	21.10 (19.7–23.4)	22.10 (18.8–24.0)	22.40 (19.7–25.3)	20.80 (19.2–21.1)	20.65 (19.8–22.8)	22.55 (18.6–24.4)	23.00 (21.4–24.6)	22.75 (19.8–27.9)

BMI, body mass index; CSF, cerebrospinal fluid; max, maximum; min, minimum.

Table S2. Demographics and baseline characteristics of individuals with NT1 in the single-rising-dose study

	Individuals with NT1			
	**Placebo[a] (*n* = 13)**	**danavorexton 5 mg (*n* = 6)**	**danavorexton 11.2 mg (*n* = 4)**	**danavorexton 44.8 mg (*n* = 4)**
Age, years				
Median (min–max)	34.0 (20–59)	36.0 (20–59)	34.5 (20–39)	23.5 (18–44)
Sex, *n* (%)				
Male	6 (46.2)	2 (33.3)	2 (50.0)	2 (50.0)
Female	7 (53.8)	4 (66.7)	2 (50.0)	2 (50.0)
BMI, kg/m^2				
Median (min–max)	24.10 (20.3–39.4)	27.85 (21.2–34.3)	23.60 (20.3–32.6)	25.80 (22.8–39.4)
MWT score				
Median (min–max)	2.50 (0.0–15.0)	1.75 (0.0–11.5)	8.00 (0.5–15.0)	3.50 (1.5–6.0)
ESS score				
Median (min–max)	18.0 (10–20)	13.5 (10–19)	18.5 (14–20)	18.5 (14–20)
Age of onset (years)				
Median (min–max)	15.0 (10–50)	16.5 (10–50)	22.5 (13–36)	14.5 (12–16)
Time since diagnosis, years				
Median (min–max)	9.0 (1–38)	10.0 (5–38)	4.5 (1–24)	9.5 (4–29)
Cataplexy episodes per week for the past month,[b] *n*				
Mean (SD)	3.7 (3.92)	3.7 (5.09)	3.5 (3.11)	5.5 (4.20)
Naps per day for the past month,[b] *n*				
Median (min–max)	1.0 (0–4)	0.5 (0–2)	2.5 (0–4)	1.5 (0–3)
Amount of sleep per day for the past month,[b] minutes				

Median (min–max)	360.0 (270–480)	420.0 (300–480)	330.0 (300–450)	360.0 (270–450)

[a]All participants in the placebo group also received a dose of danavorexton because of the crossover study design. [b]Based on self-report during the past month (not including the washout period from at least 7 days before to the start of the study). BMI, body mass index; ESS, Epworth Sleepiness Scale; max, maximum; min, minimum; MWT, Maintenance of Wakefulness Test; NT1, narcolepsy type 1; SD, standard deviation.

Table S3. Demographics and baseline characteristics of healthy volunteers, individuals with NT1 and individuals with NT2 in the multiple-rising-dose study

	Healthy volunteers				Adults with NT1			Adults with NT2		
	Placebo	danavorexton			Placebo	danavorexton		Placebo	danavorexton	
	(*n* = 6)	44 mg (IV) (*n* = 6)	112 mg (IV) (*n* = 6)	180 mg (IV) (*n* = 6)	(*n* = 4)	11 mg (IV) (*n* = 4)	44 mg (IV) (*n* = 5)	(*n* = 5)	44 mg (IV) (*n* = 4)	112 mg (IV) (*n* = 5)
Age, years										
Median (min–max)	24.0 (20–30)	23.0 (21–30)	22.5 (21–30)	22.0 (22–23)	28.0 (18–39)	37.5 (34–43)	26.0 (20–40)	24.0 (24–48)	30.5 (23–41)	23.0 (20–35)
Sex, *n* (%)										
Male	6 (100.0)	6 (100.0)	6 (100.0)	6 (100.0)	3 (75.0)	2 (50.0)	3 (60.0)	2 (40.0)	1 (25.0)	3 (60.0)
Female	0	0	0	0	1 (25.0)	2 (50.0)	2 (40.0)	3 (60.0)	3 (75.0)	2 (40.0)
BMI, kg/m^2										
Median (min–max)	22.20 (21.3–23.9)	21.10 (19.9–22.8)	22.00 (20.4–24.7)	22.05 (19.3–24.8)	24.85 (22.4–27.2)	28.25 (20.5–32.3)	25.90 (17.3–28.8)	19.80 (17.8–26.0)	24.40 (16.6–25.9)	20.60 (19.2–28.0)
MWT score (sleep latency)										
Median (min–max)	NA	NA	NA	NA	1.81 (1.1–3.1)	2.25 (1.0–2.6)	4.50 (0.5–10.9)	3.13 (0.9–9.0)	5.50 (3.8–11.3)	4.38 (3.0–17.4)
ESS score										
Median (min–max)	NA	NA	NA	NA	17.5 (10–21)	17.5 (13–24)	18.0 (13–23)	17.0 (14–22)	17.5 (14–19)	18.0 (15–21)
Age of onset, years										

Median (min–max)	NA	NA	NA	NA	14.5 (11–23)	23.0 (14–36)	13.0 (7–15)	14.0 (14–20)	16.5 (15–18)	16.0 (12–23)
Time since diagnosis, years										
Median (min–max)	NA	NA	NA	NA	12.5 (4–21)	15.5 (3–22)	14.0 (5–26)	10.0 (4–34)	13.5 (6–26)	6.0 (0–23)
Patient-reported cataplexy events per week,[a] *n*										
Mean (SD)	NA	NA	NA	NA	6.3 (5.19)	11.3 (10.01)	3.6 (2.70)	–	–	–
Patient-reported number of naps (brief episodes of sleep) during the day, per week,[a] *n*										
Median (min–max)	NA	NA	NA	NA	12.0 (7–33)	10.5 (7–18)	10.0 (4–22)	7.0 (1–12)	4.5 (0–8)	6.0 (3–14)
Total sleep time per day at baseline, minutes										
Median (min–max)	NA	NA	NA	NA	405.0 (285–480)	420.0 (420–480)	420.0 (300–420)	465.0 (390–480)	420.0 (375–465)	420.0 (360–480)

[a]Based on self-report during the past month (not including the washout period from at least 7 days before to the start of the study). BMI, body mass index; ESS, Epworth Sleepiness Scale; IV, intravenous; max, maximum; min, minimum; MWT, Maintenance of Wakefulness Test; NA, not applicable; NT1, narcolepsy type 1; NT2, narcolepsy type 2; SD, standard deviation.

Table S4. TEAEs per dose group in healthy adults and individuals with NT1 in the single-rising-dose study

	Healthy adults											Healthy elderly		Individuals with NT1			
	Cohorts 1 and 2							Supplementary cohorts S1 and S2			CSF						
**Preferred term**	**Placebo (*n* = 11)**	**danavorexton 7 mg (*n* = 6)**	**danavorexton 14 mg (*n* = 6)**	**danavorexton 28 mg (*n* = 5)**	**danavorexton 56 mg (*n* = 6)**	**danavorexton 112 mg (*n* = 6)**	**danavorexton 134.4 mg (n = 6)**	**Placebo (*n* = 4)**	**danavorexton 180 mg (*n* = 6)**	**danavorexton 240 mg (*n* = 6)**	**danavorexton 112 mg (*n* = 4)**	**Placebo (*n* = 2)**	**danavorexton 112 mg (*n* = 6)**	**Placebo (*n* = 13)**	**danavorexton 5 mg (*n* = 6)**	**danavorexton 11.2 mg (*n* = 4)**	**danavorexton 44.8 mg (*n* = 4)**
Participants with any TEAEs, *n* (%)	1 (9.1)	1 (16.7)	1 (16.7)	1 (20.0)	1 (16.7)	1 (16.7)	2 (33.3)	1 (25.0)	3 (50.0)	5 (83.3)	1 (25.0)	–	3 (50.0)	–	–	1 (25.0)	4 (100.0)
Nausea	–	–	–	–	–	–	1 (16.7)	–	–	–	–	–	–	–	–	–	–
Feeling drunk	–	–	–	–	–	–	1 (16.7)	–	–	–	–	–	–	–	–	–	–
Influenza	–	1 (16.7)	–	–	–	–	–	–	–	–	–	–	–	–	–	–	–
Nasopharyngitis	–	–	–	–	–	–	–	–	–	1 (16.7)	–	–	–	–	–	–	–
Blood pressure increased	–	–	–	–	–	–	2 (33.3)	–	2 (33.3)	4 (66.7)	–	–	3 (50.0)	–	–	–	1 (25.0)
Electrocardiogram PR prolongation	–	–	–	1 (20.0)	–	1 (16.7)	–	–	–	–	–	–	–	–	–	–	–
Heart rate increased	–	–	–	–	–	–	2 (33.3)	–	–	–	–	–	–	–	–	–	1 (25.0)
Blood triglycerides increased	–	–	–	–	1 (16.7)	–	–	–	–	–	–	–	–	–	–	–	–
White blood cell count	1 (9.1)	–	–	–	–	–	–	–	–	–	–	–	–	–	–	–	–
C-reactive protein increased	–	–	–	–	–	–	–	–	–	1 (16.7)	–	–	–	–	–	–	–
Oropharyngeal pain	–	–	1 (16.7)	–	–	–	–	–	–	–	–	–	–	–	–	–	–
Dizziness	–	–	–	–	–	–	–	–	1 (16.7)	–	–	–	–	–	–	–	–

Orthostatic hypotension	–	1 (16.7)	–	–	–	–	–	1 (25.0)	1 (16.7)	–	–	–	–	–	–	–	–
Salivary hypersecretion	–	–	–	–	–	–	–	–	–	–	–	–	–	–	–	–	1 (25.0)
Pharyngitis	–	–	–	–	–	–	–	–	–	–	–	–	–	–	–	–	1 (25.0)
Narcolepsy	–	–	–	–	–	–	–	–	–	–	–	–	–	–	–	1 (25.0)	–
Euphoric mood	–	–	–	–	–	–	–	–	–	–	–	–	–	–	–	–	1 (25.0)
Hypnagogic hallucination	–	–	–	–	–	–	–	–	–	–	–	–	–	–	–	–	1 (25.0)
Logorrhea	–	–	–	–	–	–	–	–	–	–	–	–	–	–	–	–	1 (25.0)
Cough	–	–	–	–	–	–	–	–	–	–	–	–	–	–	–	–	1 (25.0)
Back pain											1 (25.0)	–	–				

CSF, cerebrospinal fluid; NT1, narcolepsy type 1; TEAE, treatment-emergent adverse event.

Table S5. TEAEs per dose group in healthy adults, individuals with NT1 and individuals with NT2 in the multiple-rising-dose study

Preferred term	Number of healthy participants *n* (%)				Number of adults with NT1 *n* (%)			Number of adults with NT2 *n* (%)		
	Placebo (*n* = 6)	danavorexton 44 mg (*n* = 6)	danavorexton 112 mg (*n* = 6)	danavorexton 180 mg (*n* = 6)	Placebo (*n* = 4)	danavorexton 11 mg (*n* = 4)	danavorexton 44 mg (*n* = 5)	Placebo (*n* = 5)	danavorexton 44 mg (*n* = 4)	danavorexton 112 mg (*n* = 5)
Participants with any TEAEs, *n* (%)	1 (16.7)	–	2 (33.3)	4 (66.7)	1 (25.0)	1 (25.0)	5 (100.0)	–	1 (25.0)	3 (60.0)
Diarrhea	–	–	–	–	–	–	–	–	1 (25.0)[a]	–
Nausea	–	–	–	–	–	–	1 (20.0)[a]	–	1 (25.0)	–
Salivary hypersecretion	–	–	–	1 (16.7)[a]	–	–	1 (20.0)	–	–	1 (20.0)[a]
Feeling abnormal	–	–	1 (16.7)[a]	1 (16.7)[a]	–	–	–	–	–	–
Asthenia	–	–	–	1 (16.7)[a]	–	–	–	–	–	–
Gastroenteritis	–	–	–	–	–	–	–	–	–	1 (20.0)
Nasopharyngitis	–	–	1 (16.7)	1 (16.7)	–	–	–	–	–	–
Headache	–	–	–	–	–	1 (25.0)	–	–	–	–
Pollakiuria	–	–	–	–	–	–	4 (80.0)[a]	–	–	2 (40.0)[a]
Dysmenorrhea	–	–	–	–	–	–	1 (20.0)	–	–	–
Dermatitis contact	1 (16.7)	–	–	–	1 (25.0)	–	–	–	–	–
Hyperhidrosis	–	–	–	–	–	–	1 (20.0)[a]	–	–	–
Hot flush	–	–	–	1 (16.7)[a]	–	–	–	–	–	–
Blood pressure increased	–	–	–	–	–	–	–	–	–	1 (20.0)[a]

[a]Considered by the investigator to be related to the study drug. NT1, narcolepsy type 1; NT2, narcolepsy type 2; TEAE, treatment-emergent adverse event.

Table S6. Summary of PK parameters of danavorexton after a single IV infusion in healthy adult participants, healthy elderly participants and individuals with NT1 in the single-rising-dose study

		Healthy adults								Healthy elderly	Individuals with NT1		
**PK parameter (unit)**	**Descriptive statistic**	**danavorexton 7 mg**	**danavorexton 14 mg**	**danavorexton 28 mg**	**danavorexton 56 mg**	**danavorexton 112 mg**	**danavorexton 134.4 mg**	**danavorexton 180 mg**	**danavorexton 240 mg**	**danavorexton 112 mg**	**danavorexton 5 mg**	**danavorexton 11.2 mg**	**danavorexton 44.8 mg**
C_{max} (ng/mL)	*n*	5	6	5	6	6	6	6	6	6	5	4	4
	Geometric mean	16.70	36.83	57.56	122.6	235.2	304.9	344.5	506.3	278.4	10.10	21.86	96.48
	%CV	17.1	11.9	14.2	10.6	17.1	16.4	6.8	17.0	11.8	19.1	11.5	22.4
C_{eoi} (ng/mL)	*n*	5	6	5	6	6	6	6	6	6	5	4	4
	Geometric mean	13.37	29.81	52.08	114.6	213.8	290.3	324.0	493.2	272.2	9.649	21.17	94.04
	%CV	14.9	14.2	13.8	11.6	14.7	11.0	9.3	14.1	13.5	11.7	8.4	24.6
t_{max} (h)	*n*	5	6	5	6	6	6	6	6	6	5	4	4

	Median	3.000	4.000	3.000	4.000	4.000	6.000	6.500	4.000	6.500	9.000	9.000	6.500
	Min, max	2.00, 4.00	3.00, 4.00	2.00, 4.00	3.00, 9.00	1.50, 9.00	3.00, 9.00	3.00, 9.00	3.00, 9.00	1.50, 9.00	4.00, 9.00	4.00, 9.00	4.00, 9.00
AUC_∞ (ng*h/mL)	*n*	5	6	5	6	6	6	6	6	6	4	4	4
	Geometric mean	141.8	328.1	523.5	1065	2092	2682	3013	4585	2590	88.45	200.7	925.3
	%CV	15.9	15.4	16.7	17.4	15.1	19.1	5.3	14.9	10.9	15.3	6.6	30.9
CL (l/h)	*n*	5	6	5	6	6	6	6	6	6	4	4	4
	Mean	50.00	43.07	54.20	53.10	54.10	50.90	59.83	52.78	43.50	57.05	55.90	50.18
	SD	9.0785	6.4856	9.9541	8.2202	9.0437	9.8018	3.3381	7.4882	4.9651	9.5577	3.7692	15.533
$t_{1/2z}$ (h)	*n*	5	6	5	6	6	6	6	6	6	4	4	4
	Mean	4.120	4.498	5.142	3.860	3.788	3.943	3.363	4.033	4.345	3.465	3.923	4.130
	SD	1.9190	1.7725	1.8060	0.6478	1.0424	0.9492	0.8523	1.2843	0.9501	1.0998	1.0265	1.1981

AUC_∞, area under the plasma concentration–time curve from time 0 to infinity; C_{eoi}, concentration at the end of infusion; CL, total clearance; C_{max}, maximum observed concentration; CV, coefficient of variance; IV, intravenous; max, maximum; min, minimum; NT1, narcolepsy type 1; PK, pharmacokinetic; SD, standard deviation; $t_{1/2z}$, terminal disposition phase half-life; t_{max}, time of first occurrence of C_{max}.

Table S7. Summary of PK parameters of danavorexton given as a 9-hour IV infusion in healthy volunteers, individuals with NT1 and individuals with NT2 in the multiple-rising-dose study

	Healthy volunteers			NT1		NT2	
**PK parameter**	**danavorexton 44 mg (*n* = 6)**	**danavorexton 112 mg (*n* = 6)**	**danavorexton 180 mg (*n* = 6)**	**danavorexton 11 mg (*n* = 4)**	**danavorexton 44 mg (*n* = 5)**	**danavorexton 44 mg (*n* = 4)**	**danavorexton 112 mg (*n* = 5)**
Day 1							
AUC_∞ (h*ng/mL) Geometric mean (%CV)	670.0 (11.5)	1527 (10.4)	2639 (15.1)	165.9 (11.6)	687.9 (18.2)	651.2 (12.2)	1500 (18.7)
C_{eoi} (ng/mL) Geometric mean (%CV)	74.49 (5.8)	166.9 (12.5)	300.2 (16.6)	17.39 (7.4)	70.20 (26.1)	68.74 (10.7)	156.1 (18.5)
$t_{1/2z}$ (h) Mean (SD)	3.950 (1.0651)	3.712 (1.0380)	3.707 (0.8872)	3.300 (1.3149)	3.362 (0.5036)	2.708 (0.2133)	2.800 (0.6642)
CL (l/h) Mean (SD)	66.02 (7.0033)	73.73 (7.3189)	68.92 (11.253)	66.73 (7.8390)	64.78 (11.263)	68.00 (8.9870)	75.66 (13.241)
Day 7							
AUC_τ (h*ng/mL) Geometric mean (%CV)	650.7 (11.9)	1523 (11.4)	2747 (13.5)	182.7 (12.3)	722.7 (14.0)	657.1 (11.0)	1519 (16.2)
C_{min} (ng/mL) Geometric mean (%CV)	0.6785 (72.6)	1.445 (78.2)	3.455 (38.1)	0.4061 (79.9)	1.246 (38.4)	0.4542 (58.1)	1.192 (69.2)
C_{eoi} (ng/mL) Geometric mean (%CV)	70.31 (11.9)	159.2 (14.7)	295.5 (15.5)	17.68 (9.7)	73.20 (20.1)	70.87 (10.5)	153.9 (16.0)
$t_{1/2z}$ (h) Mean (SD)	3.347 (0.6552)	3.600 (0.9919)	3.338 (0.5332)	3.595 (1.2100)	4.222 (0.7716)	2.643 (0.3625)	3.136 (0.7548)
CL (l/h) Mean (SD)	68.02 (7.8316)	74.07 (9.6513)	66.08 (9.5757)	60.60 (7.5242)	61.40 (8.9392)	67.25 (6.8927)	74.46 (11.332)

AUC_∞, area under the plasma concentration–time curve from time 0 to infinity; AUC_τ area under the plasma concentration–time curve during a dosing interval; C_{eoi}, concentration at the end of infusion; C_{min}, minimum observed concentration during a dosing interval; CL, total clearance; CV, coefficient of variation; IV, intravenous; NT1, narcolepsy type 1; NT2, narcolepsy type 2; PK, pharmacokinetic; SD, standard deviation; $t_{1/2z}$, terminal disposition phase half-life.

Table S8. Concomitant medications of individuals with NT1 stopped prior to baseline in the single-rising-dose study

**Medication name, *n* (%)**	**Individuals with NT1 (*n* = 14)**
Clomipramine hydrochloride	9 (64.3)
Modafinil	9 (64.3)
Methylphenidate hydrochloride	8 (57.1)
Zolpidem tartrate	3 (21.4)
Brotizolam	2 (14.3)
Clonazepam	2 (14.3)
Pemoline	2 (14.3)
Caffeine	1 (7.1)
Chlorpromazine hydrochloride	1 (7.1)
Quetiapine fumarate	1 (7.1)
Venlafaxine hydrochloride	1 (7.1)

NT1, narcolepsy type 1.

Table S9. Concomitant medications of individuals with NT1 and individuals with NT2 stopped prior to the multiple-rising-dose study

**Medication name, *n* (%)**	**Individuals with NT1 (*n* = 13)**	**Individuals with NT2 (*n* = 14)**
Modafinil	9 (69.2)	9 (64.3)
Methylphenidate hydrochloride	7 (53.8)	1 (7.1)
Clomipramine hydrochloride	5 (38.5)	–
Pemoline	2 (15.4)	4 (28.6)
Zolpidem tartrate	2 (15.4)	–
Brotizolam	1 (7.7)	–
Clonazepam	1 (7.7)	–
Eszopiclone	1 (7.7)	–
Other cold preparations	1 (7.7)	–
Paroxetine hydrochloride hemihydrate	1 (7.7)	–
Paracetamol	–	2 (14.3)
Baloxavir marboxil	–	1 (7.1)
Bromfenac sodium	–	1 (7.1)
Chlorphenamine maleate; dihydrocodeine phosphate; methylephedrine hydrochloride-DL	–	1 (7.1)
Etodolac	–	1 (7.1)
Gatifloxacin sesquihydrate	–	1 (7.1)
Levofloxacin	–	1 (7.1)

NT1, narcolepsy type 1; NT2, narcolepsy type 2.

SI References

1. H. Yukitake *et al.*, TAK-925, an orexin 2 receptor-selective agonist, shows robust wake-promoting effects in mice. *Pharmacol Biochem Behav* **187**, 172794 (2019).
2. T. E. Scammell, J. T. Willie, C. Guilleminault, J. M. Siegel, International Working Group on Rodent Models of Narcolepsy, A consensus definition of cataplexy in mouse models of narcolepsy. *Sleep* **32**, 111–116 (2009).
3. E. L. Clark, C. R. Baumann, G. Cano, T. E. Scammell, T. Mochizuki, Feeding-elicited cataplexy in orexin knockout mice. *Neuroscience* **161**, 970–977 (2009).
4. Y. Oishi *et al.*, Role of the medial prefrontal cortex in cataplexy. *J Neurosci* **33**, 9743–9751 (2013).
5. ICH, Guideline for Good Clinical Practice E6 (R2). Available from: https://www.ema.europa.eu/en/ich-e6-r2-good-clinical-practice. (accessed December 2019). (2016).
6. M. Takegami *et al.*, Development of a Japanese version of the Epworth Sleepiness Scale (JESS) based on item response theory. *Sleep Med* **10**, 556-565 (2009).
7. FDA Department of Health and Human Services, Estimating the maximum safe starting dose in initial clinical trials for therapeutics in adult healthy volunteers. Available from: https://www.fda.gov/regulatory-information/search-fda-guidance-documents/estimating-maximum-safe-starting-dose-initial-clinical-trials-therapeutics-adult-healthy-volunteers. (accessed December 2019). (2005).
8. M. M. Mitler, K. S. Gujavarty, C. P. Browman, Maintenance of wakefulness test: a polysomnographic technique for evaluation treatment efficacy in patients with excessive somnolence. *Electroencephalogr Clin Neurophysiol* **53**, 658–661 (1982).
9. Y. Dauvilliers *et al.*, Pitolisant versus placebo or modafinil in patients with narcolepsy: a double-blind, randomised trial. *Lancet Neurol* **12**, 1068-1075 (2013).
10. T. Akerstedt, M. Gillberg, Subjective and objective sleepiness in the active individual. *Int J Neurosci* **52**, 29–37 (1990).
